# Proteomic Analysis Reveals Major Proteins and Pathways That Mediate the Effect of 17-β-Estradiol in Cell Division and Apoptosis in Breast Cancer MCF7 Cells

**DOI:** 10.1021/acs.jproteome.4c00102

**Published:** 2024-10-11

**Authors:** Zhenqi Zhou, Brihget Sicairos, Jianhong Zhou, Yuchun Du

**Affiliations:** Department of Biological Sciences, University of Arkansas, Fayetteville, Arkansas 72701, United States

**Keywords:** Quantitative proteomics, proteome profiling, SILAC, estrogen, breast cancer, cell division, apoptosis

## Abstract

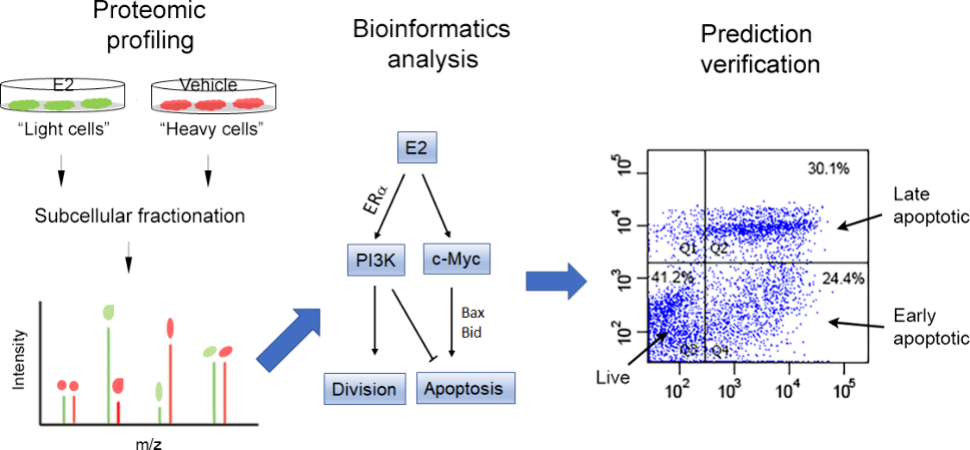

Despite extensive research, the genes/proteins and pathways responsible for the physiological effects of estrogen remain elusive. In this study, we determined the effect of estrogen on global protein expression in breast cancer MCF7 cells using a proteomic method. The expression of 77 cytosolic, 74 nuclear, and 81 membrane/organelle proteins was significantly altered by 17-β-estradiol (E2). Protein enrichment analyses suggest that E2 may stimulate cell division primarily by promoting the G1 to S phase transition and advancing the G2/M checkpoint. The effect of E2 on cell survival was complex, as it could simultaneously enhance and inhibit apoptosis. Bioinformatics analysis suggests that E2 may enhance apoptosis by promoting the accumulation of the pore-forming protein Bax in the mitochondria and inhibit apoptosis by activating the PI3K/AKT/mTOR signaling pathway. We verified the activation of the PI3K signaling and the accumulation of Bax in the membrane/organelle fraction in E2-treated cells using immunoblotting. Treatment of MCF7 cells with E2 and the PI3K inhibitor Ly294002 significantly enhanced apoptosis compared to those treated with E2 alone, suggesting that combining estrogen with a PI3K inhibitor could be a promising strategy for treating ERα-positive breast cancer. Interestingly, many of the E2-upregulated proteins contained the HEAT, KH, and RRM domains.

## Introduction

Estrogen plays an essential role in the proliferation and differentiation of normal breast tissue and is linked to the development of breast cancer. The main form of estrogen in the cells is 17-β-estradiol (E2). E2 stimulates breast cell proliferation by binding to estrogen receptor alpha (ERα) thereby altering estrogen-responsive gene expression that regulates the cell cycle and apoptosis.^1,2^ E2 can influence cell behavior through genomic and nongenomic actions. In the genomic mechanism, the E2-bound ERα translocates into the nucleus, where it binds estrogen response elements (EREs) and regulates the expression of estrogen-responsive genes.^3^ Alternatively, ERα can also regulate gene expression by interacting with other transcription factors such as AP1.^4,5^ In the nongenomic mechanism, E2 binds the plasma membrane-bound ERα and the G-protein-coupled receptor GPR30 leading to rapid activation of several signaling pathways.^3^

The effect of estrogen on cell survival is complex. On the one hand, estrogen can induce apoptosis in long-term estrogen-deprived (LTED) cells or in cells treated exhaustively with antiestrogen by activating mitochondrion-dependent and receptor-activated pathways, such as upregulation of cytochrome c and Fas/FasL.^6^ On the other hand, estrogen has been shown to inhibit apoptosis by activating the phosphoinositide 3-kinase (PI3K) signaling pathway via the binding of ERα to the p85α regulatory subunit of PI3K^7^ and inducing the expression of antiapoptotic genes.^8^

For most ERα-positive breast cancer patients, endocrine therapy is the first choice of treatment, which includes the use of selective ER modulators (SERMs; e.g., tamoxifen and raloxifene), selective ER down-regulators (SERDs; e.g., fulvestrant), and/or aromatase inhibitors (e.g., anastrozole); all of which disrupt E2 signaling through the disruption of E2-ERa binding or by inhibiting E2 production.^9^ Although the antiestrogen drugs tamoxifen and raloxifene have been successfully used to treat ERα-positive breast cancer for decades, approximately 40% of patients treated with antiestrogen therapy for five to ten years develop resistance toward tamoxifen or raloxifene and 50% of women with metastatic breast cancer do not respond to antiestrogen treatment,^10^ creating a major issue in the treatment of ERα-positive breast cancers. Because E2 can induce apoptosis, E2 was successfully used in high concentrations to treat ERα-positive breast cancer in postmenopausal women^11^ before tamoxifen was used as an antiestrogen drug.^12^ After the development of antiestrogen drugs, E2 has been exploited as an alternative drug to overcome antiestrogen resistance in the treatment of ER-positive breast cancer.^13,14^ Although the results are not conclusive, there is potential for E2 as a viable therapy for breast cancer patients.^15^

Due to the profound biological and pathological effects of estrogen on normal and breast cancer cells, researchers have undertaken extensive efforts to identify ERα target genes associated with its physiological effects. DNA microarray and RNA sequencing analyses, for instance, have revealed the impact of estrogen on the expression of hundreds of genes.^16−20^ Additionally, chromatin immunoprecipitation (Chip)-sequencing and Chip-microarray analyses have uncovered thousands of ERα-binding sites in breast cancer cells.^21−24^ Moreover, proteomic methods have been employed to identify ERα-regulated proteins in breast cancer cells.^25^ Despite these efforts, the genes and pathways responsible for the physiological effects of estrogen remain elusive.

To gain a better understanding of the key proteins and pathways that mediate the global effects of estrogen in breast cancer cells, we employed a SILAC (Stable Isotope Labeling by Amino Acids in Cell Culture)-based quantitative proteomic method^26,27^ coupled with subcellular fractionation and subsequent bioinformatics analysis to assess the major pathways affected by E2 in human breast cancer MCF7 cells—a model widely used for studying hormone response in breast cancer.^5,17,18,24^ Our results suggest that E2 may promote cell division primarily by enhancing the G1 to S phase transition and advancing the G2/M checkpoint. Moreover, our bioinformatics analysis suggests that E2 has a dual effect on apoptosis, enhancing and inhibiting apoptosis simultaneously. By leveraging the dual effect of estrogen on apoptosis, we tested the possibility of enhancing the pro-apoptotic effect of estrogen by blocking the inhibitory pathway identified in this study. Our findings suggest that coadministration of estrogen and a PI3K inhibitor could be a promising strategy for treating ERα-positive breast cancer.

## Materials and Methods

### Cell Culture, Proteome Labeling, and E2 Treatment

The MCF7 cell line was maintained in α-MEN with 5% FBS, 100 units/ml penicillin, and 100 μg/mL streptomycin at 37 °C in a 5% CO_2_ atmosphere. For proteome labeling, the MCF7 cells were cultured in unlabeled α-MEN with 5% dialyzed FBS, 100 units/ml penicillin, and 100 μg/mL streptomycin (light medium) or the α-MEN containing arginine-^13^C_6_ and lysine-^13^C_6_^15^N_2_ with 5% dialyzed FBS, 100 units/ml penicillin, and 100 μg/mL streptomycin (heavy medium) for 2 weeks. The unlabeled and the labeled cells were then cultured in the unlabeled and labeled, phenol red-free α-MEN with charcoal treated 5% dialyzed FBS, 100 units/ml penicillin, and 100 μg/mL streptomycin for 6 days. After the 6-day hormonal starvation, the cells in the unlabeled medium were treated with 100 nM E2 (Sigma, St. Louis, MO), and the cells in the labeled medium were treated with an equivalent amount of vehicle (ethanol) for 18 h.

### Subcellular Fractionation

After the E2 (or ethanol for control) treatment, the cells were harvested and washed twice with cold phosphate-buffered saline (PBS). The cells were then resuspended in 5 packed cell pellet volumes of hypotonic buffer [20 mM Hepes-NaOH, pH 7.5, 10 mM KCl, 1.5 mM MgCl_2_, 250 mM sucrose, 1 mM DTT, and protease inhibitor mixture (Roche Applied Science)], incubated on ice for 30 min, and lysed with approximately 20 strokes of a tight-fitting pestle (type B) in Dounce homogenizer (Kontes Glass Co., Vineland, NJ) until >95% of cells were ruptured. The cell lysate was then centrifuged at 1,000 × g at 4 °C for 10 min, and the pellet was collected as the nuclear fraction. The supernatant was centrifuged at 10,000 × g at 4 °C for 15 min. The resulting supernatant was designated as the cytosolic protein, and the pellet was collected as the membrane/organelle fraction. The nuclear fraction and the membrane/organelle fraction were then respectively dissolved in a modified RIPA buffer (50 mM Hepes-NaOH, pH 7.5, 150 mM NaCl, 1.5 mM MgCl_2_, 1 mM EGTA, 10% glycerol, 1% Triton X-100, 1% SDS, and protease inhibitor mixture). After centrifugation at 21,000 × g at 4 °C for 10 min, the resulting supernatant from the nuclear fraction was designated as nuclear protein, and the supernatant from the membrane/organelle fraction was designed as membrane/organelle protein. The concentrations of the cytosolic, nuclear, and membrane/organelle proteins were determined by the Bio-Rad RC DC method (Hercules, CA). It is important to note that the subcellular fractionation procedures were designed for simplicity and consistency, with the goal of increasing the coverage of the proteomes rather than accurately characterizing the subproteomes.

### Gel Electrophoresis, in-Gel Digestion, and LC-MS/MS

Equal amounts of unlabeled cytosolic, nuclear, and membrane/organelle proteins were mixed separately with equal amounts of labeled cytosolic, nuclear, and membrane/organelle proteins. The mixed protein was then fractionated with a 12% SDS-PAGE gel, and the fractioned proteins were visualized by Coomassie brilliant blue G-250. Each protein lane of the SDS-PAGE gel was cut into 13–15 slices. Proteins in gel slices were digested with trypsin (Promega, Madison, WI) overnight at 37 °C and the resulting peptides were dissolved in 20 μL 0.1% formic acid and then analyzed with an LTQ Orbitrap XL mass spectrometer (ThermoFisher Scientific, Waltham, MA) operated in a data-dependent mode for tandem MS. The LC-MS/MS analysis was performed as previously described in detail.^28^

### Protein Identification and Quantification

Raw data from the LC-MS/MS analysis were processed by MaxQuant (version 1.6.2.10)^29^ with the built-in search engine Andromeda and searched against a target-decoy human SwissProt protein database (20,408 entries) retrieved from UniProt (www.uniprot.org) as described previously.^28,30^ Specifically, the false discovery rates (FDRs) for peptide and protein identification were set to 1%. The MS error tolerance was set to 4.5 ppm, and the MS/MS error tolerance was set to 20 ppm. The minimum required peptide length was set to 7 amino acids, and a maximum of 2 missed cleavages was allowed. Variable modifications of acetylation at peptide N-terminus and oxidation on methionine, and fixed modification of cysteine carbamidomethylation were included. SILAC ratios (light/heavy ratios) were calculated using unique and razor peptides with a minimum ratio of 2.

The proteins matched to the reverse database, identified only by site or single peptide, and common contaminants were removed. The remaining proteins were analyzed by Perseus (version 2.0.3.0),^31^ and the Significance B score was obtained for the quantified proteins. The Significance B score is a significance score for protein SILAC ratios and identifies outliers based on the standard deviation of the protein SILAC ratios of the main distribution and signal intensity.^32^ A protein was considered a differentially expressed protein when its ratio was significant by the Significance B score with a *p* < 0.05, and the L/H ratio was >1.5 or <0.65.

### Bioinformatics Analysis of the E2-Regulated Proteins

The cytosolic, nuclear, and membrane/organelle proteins that were identified and quantified with at least two peptides were separately analyzed with GSEA using the module of PreRanked genes.^33^ The proteins were first analyzed using the Hallmark gene collection and then the Pathway Interaction Database (PID) to identify the pathways affected by the E2 treatment. A gene set with an FDR < 0.25, which was the preferred criterion for GSEA analysis,^33^ was considered enriched. The E2-regulated proteins (up or downregulated) were also analyzed by DAVID, a web server for functional annotation and enrichment analyses of gene lists.^34^ For the DAVID analysis, the differentially expressed cytosolic, nuclear, and membrane/organelle proteins were pooled together. During the pooling process, proteins identified in two or more fractions (i.e., shared proteins) with conflicting SILAC ratios (e.g., one up and others down) were removed from the pooled protein list. In the DAVID enrichment analysis, the E2-regulated proteins were cluster-analyzed with GOTERM_BP_DIRECT and GOTERM_MF_DIRECT to determine the functional enrichment within the pooled list of proteins. The biological pathways and molecular function categories with *p* < 0.05 were considered significant. The E2-regulated proteins were also cluster-analyzed with UP_SEQ_FEATURE to determine the sequence features of the E2-regulated proteins. The E2-regulated (up or downregulated) proteins were further loaded to FunRich^35^ to identify the enriched biological pathways and cellular compartments. Biological pathways and cellular compartments with *p* < 0.05 were considered significantly enriched.

### Western Blotting

After culturing MCF7 cells in phenol red-free α-MEM with charcoal-treated 5% dialyzed FBS, 100 units/ml penicillin, and 100 μg/mL streptomycin for 3 days, the cells were treated with E2 to detect AKT Ser-473 phosphorylation or Bax protein levels using Western blotting. For the detection of AKT Ser-473 phosphorylation, the estrogen-starved cells were either mock-treated or treated with 10 nM E2 for 10 min. Subsequently, the cells were harvested, lysed, and the resulting proteins were fractionated on a 4–12% Bis-Tris NuPAGE gel under reducing conditions. Western blotting was then performed according to the manufacturer’s instructions (Thermo Fisher Scientific, Waltham, MA). To determine Bax protein levels in the membrane/organelle fractions, the estrogen-starved cells were either mock-treated or treated with 100 nM E2 for 18 h. Following treatment, the cells were harvested, and membrane/organelle proteins were prepared as described above for LC-MS/MS analysis. The resulting membrane/organelle proteins were then analyzed using Western blotting as previously described.^30,36^ The Western blot images were acquired using a Li-COR 9120 scanner (Li-COR Inc., Lincoln, NE). The anti-AKT (Cat#: 10176–2-AP), anti-F1-β-ATPase (Cat#: 17247–1-AP), antihistone H4 (16047–1-AP), and anti-Bax (Cat#: 50599–2-Ig) antibodies were purchased from Proteintech (Rosemont, IL). The antiphospho-AKT (Ser-473) antibody (Cat#: 9271) was from Cell Signaling (Danvers, MA), anti-EGFR (A19002A) was from Biolegend (San Diego, CA), and the antitubulin antibody (Cat#: T9026) was from Sigma (St. Louis, MO).

### Treatment of MCF7 Cells with E2 and a PI3K Inhibitor and Flow Cytometry Analysis of the Cells

MCF7 cells (2.5 × 10^5^ cells) grown in α-MEM medium supplemented with 5% charcoal-treated FBS were either vehicle-treated or treated with 25 μM PI3K inhibitor Ly294002 for 1 h. E2 was then added to the mock-treated or Ly294002-treated cells to a final concentration of 0, 0.1, 1, or 10 μM. Subsequently, the cells were incubated with the inhibitor and E2 for another 18 h. After the treatments, the cells were incubated with 0.05% trypsin, harvested, and washed twice with cold PBS. The cells were then resuspended in 0.1 mL of binding buffer (10 mM Hepes-NaOH, pH7.4, 140 mM NaCl, and 2.5 mM CaCl_2_) and stained with annexin V using a FITC Annexin V Apoptosis Detection kit I (BD Pharmingen 556547) according to the manufacturer’s instructions. Briefly, 5 μL of annexin V and 5 μL of propidium iodide were added to the cell suspension, and the mixture was incubated at room temperature in the dark for 20 min. After adding 400 μL of PBS to the cells, the cells were analyzed with a FACSAria Fusion flow cytometer (BD Biosciences, San Jose, CA). While the annexin V and PI negative cells were defined as viable cells, the annexin V positive and PI negative were defined as early apoptotic cells, and the annexin V and PI positive cells as late apoptotic cells.^37^

### Statistical Analysis

The statistical analyses for GSEA, DAVID, and FunRich enrichment analyses were conducted using the default settings in the respective software. To analyze differences in apoptosis and viability between various pairs of conditions in Figure 3, we employed a *t* test.

## Results

### Identification of E2-Induced Changes in Protein Expression in MCF7 Cells

**Figure 1:**
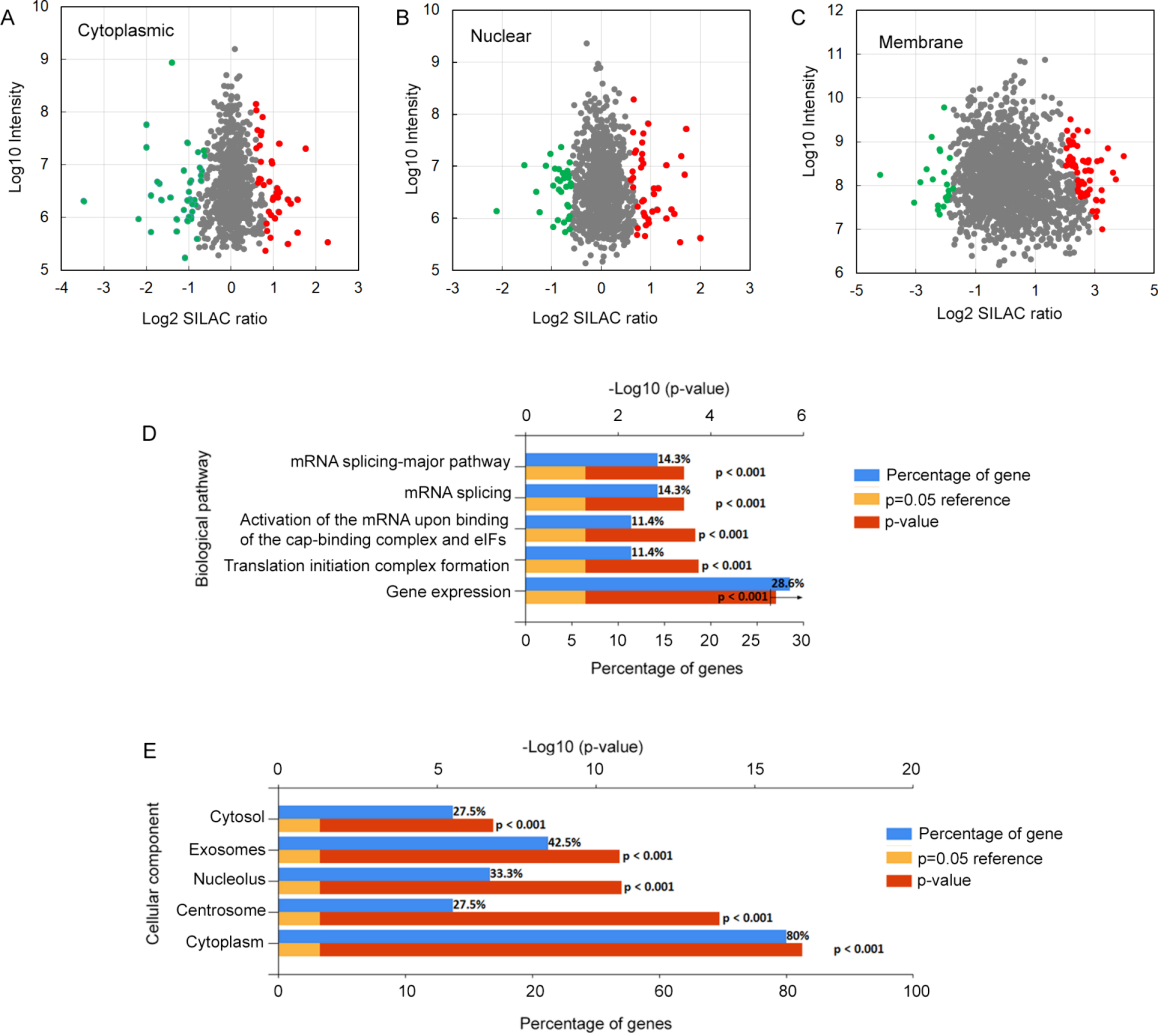
E2-regulated proteins and enrichment analysis of biological pathways and cellular components in the E2-upregulated proteins. SILAC proteomic analyses after E2 treatment and fractionation of MCF7 cells identified the upregulated (red) and downregulated proteins (green) in the cytosolic (A), nuclear (B), and membrane/organelle (C) fractions. FunRich analyses of the E2-upregulated proteins revealed the biological pathways (D) and cellular components (E) enriched in the E2-treated MCF7 cells compared to vehicle-treated control cells.

We used a SILAC-based quantitative proteomic method^26,27^ to identify the E2-regulated proteins in MCF7 cells. To increase the chances of detecting novel/low abundant proteins, we fractionated total cellular protein into the cytosolic, nuclear, and membrane/organelle fractions (Supporting Figure S1) and analyzed each fraction independently. We quantified 776 cytosolic, 936 nuclear, and 1,703 membrane/organelle proteins by at least 2 unique peptides (Supporting Tables S1–S3). Cut-offs of *p* < 0.05 in the Significance B score in Perseus analysis and an L/H fold change in SILAC ratios (E2 treated/vehicle-treated) of >1.5 or <0.65 resulted in 77 cytosolic, 74 nuclear, and 81 membrane/organelle proteins that were considered differentially expressed between the E2-treated cells and the vehicle-treated control cells (Figure 1A–C; Supporting Tables S4–S6). Some E2-regulated proteins were consistently identified in two of the three fractions. For instance, carbonic anhydrase 2 (gene name: CA2) and anterior gradient protein 3 (AGR3) were upregulated, whereas ubiquitin-like protein ISG15 (ISG15) was downregulated by E2 in both the cytosolic and the nuclear fractions. Similarly, scinderin (SCIN) exhibited downregulation by E2 in both the nuclear and the membrane/organelle fractions (Table 1). Furthermore, the expression of many of the identified E2-regulated proteins has previously been shown to be associated with breast cancer. For example, the expression of all of the top 2–4 E2-upregulated or E2-downregulated proteins (Table 1) has been shown to be associated with breast cancer,^38,39^ except for endosome/lysosome-associated apoptosis and autophagy regulator 1 (ELAPOR1), protein FAM83H (FAM83H), and pyridoxal-dependent decarboxylase domain containing 1 (PDXDC1), whose roles in breast cancer remain unreported.

**Table 1:** Short List of Top E2-Regulated Proteins in the Cytosolic, Nuclear, and Membrane/Organelle Fractions

	UniProt ID	gene name	SILAC L/H ratio	no peptide	protein name
cytosolic fraction	Q04637	EIF4G1	4.83	2	eukaryotic translation initiation factor 4 gamma 1
	P00918	CA2	3.37	4	carbonic anhydrase 2
	Q3YEC7	RABL6	2.94	2	RAB, member RAS oncogene family like 6
	Q8TD06	AGR3	2.94	3	anterior gradient 3
	P05161	ISG15	0.25	4	ISG15 ubiquitin like modifier
	P61019	RAB2A	0.22	3	RAB2A, member RAS oncogene family
	Q9Y6U3	SCIN	0.09	6	scinderin
nuclear fraction	Q6UXG2	ELAPOR1	3.99	2	endosome-lysosome associated apoptosis and autophagy regulator 1
	P38159	RBMX	3.27	10	RNA binding motif protein X-linked
	Q8TD06	AGR3	3.20	4	anterior gradient 3
	P00918	CA2	3.05	5	carbonic anhydrase 2
	Q16629	SRSF7	0.34	3	serine and arginine rich splicing factor 7
	P05161	ISG15	0.23	2	ISG15 ubiquitin like modifier
membrane/organelle fraction	Q6ZRV2	FAM83H	15.58	18	family with sequence similarity 83 member H
	O95747	OXSR1	12.99	6	oxidative stress responsive kinase 1
	Q6P996	PDXDC1	12.13	8	pyridoxal dependent decarboxylase domain containing 1
	Q15365	PCBP1	10.83	8	poly(rC)-binding protein 1
	Q9Y6U3	SCIN	0.14	7	scinderin
	Q08380	LGALS3BP	0.12	4	galectin 3 binding protein
	P32004	L1CAM	0.05	8	L1 cell adhesion molecule

### E2 Regulates the Expression of the Proteins That Affect the Transition From the G1 to S Phase and the G2/M Checkpoint

We uploaded the 776 cytosolic, 936 nuclear, and 1703 membrane/organelle proteins that were quantified by at least two unique peptides to the GSEA and analyzed the proteins using the module of PreRanked genes^33^ using the Hallmark and the PID gene collections. GSEA is a software that determines whether members of a gene set defined based on prior biological knowledge are overrepresented within the experimental data set,^33^ and the Hallmark gene collection was generated by a computational methodology identifying overlap between gene sets in other MSigDB collections to summarize well-established biological states and processes that display coherent expression among them. As expected, the “Estrogen response late” gene set was overrepresented in both the cytosolic and nuclear proteins in the GSEA analysis (Table 2). Identification of the enrichment of the estrogen response gene set in two of the three fractions serves as validation of the appropriate cellular responses to E2 treatment and our experimental procedures.

**Table 2:** List of the Gene Sets That Are Overrepresented in the E2 Treated MCF7 Cells Compared to the Vehicle-Treated Control Cells^a^

	collection: Hallmark		collection: PID pathway	
cellular fractions	enriched gene set	FDR	enriched gene set	FDR
cytosolic	estrogen response late	0.099	PID_PDGFRB pathway	0.164
			PID_VEGFR1/2 pathway	0.222
nuclear	estrogen response late	0.001		
	E2F targets	0.115		
	Heme metabolism	0.121		
	Complement	0.162		
membrane/organelle	E2F targets	0.078	PID_REG_GR pathway	0.001
	G2/M checkpoint	0.096	PID_LKB1_pathway	0.007
	PI3K AKT mTOR signaling	0.145	PID_NFAT_3 pathway	0.016
			PID_mTOR_4 pathway	0.015
			PID_Hedgehog_Gli pathway	0.015
			PID_beta_Catenin_Nuc pathway	0.014
			PID_Telomerase pathway	0.012
			PID_PDGFRB pathway	0.018
			PID_FOXO pathway	0.037
			PID_ERBB1_Downstream pathway	0.040
			PID_VEGFR1/2 pathway	0.053
			PID_MET pathway	0.060
			PID_A6B1_A6B4_Integrin pathway	0.056
			PID_ILK pathway	0.212
			PID_TRKR pathway	0.248
			PID_Thrombin_Par1 pathway	0.240
			PID_Myc_Activ pathway	0.233

a Only the gene sets with FDR less than 0.25 are listed.

In addition to the estrogen response late gene set, the GSEA analysis revealed that the “E2F targets” and “G2/M checkpoint” gene sets associated with cell cycle progression were enriched in the nuclear and/or membrane/organelle fractions (Table 2). The enrichment of the “E2F targets” and “G2/M checkpoint” gene sets in the E2-treated cells relative to vehicle-treated cells implies that the E2F transcriptional activity and G2/M checkpoint are regulated by E2 in MCF7 cells. The transition of cells from the G1 phase to the S phase is regulated at the restriction point during the late G1 phase, where cells commit to cell division if the cellular environment supports it. Beyond the restriction point, the transition from S to G2 to M to G1 becomes autonomous and no longer relies on environmental factors. Transcription of the target genes of the E2Fs is necessary for the cells to pass through the restriction point.^40^ The significance of E2F’s transcriptional activity in cell proliferation is evident from the observation that many proliferation signature genes contain binding sites for the E2Fs.^41^ The predicted regulation of E2Fs by E2 in the GSEA analysis is supported by changes in the expression of some well-known E2F target genes and proliferation signature genes.^42^ For example, the expression of minichromosome maintenance (MCM)2 and MCM7 was significantly upregulated by E2 (Supporting Table S5), while MCM3, MCM4, and MCM6 approached the significance threshold in the nuclear fraction (Supporting Table S2). The expression of MCM genes is under control of E2Fs and is critical for DNA replication in the S phase.^42,43^ Through the regulation of the expression of E2F target genes, estrogen may drive the transition from the G1 phase to the S phase of the cell cycle, facilitating DNA replication and promoting cancer cell division in estrogen-responsive breast cancers. During the G2/M checkpoint in the cell cycle, the cell undergoes checks and balances to ensure that DNA replication and cell division occur accurately. This checkpoint prevents DNA-damaged cells or the cells in which DNA replication is not completed from entering the M phase, reducing the risk of mutations. The effect of estrogen on the G2/M checkpoint has been previously reported.^44^ The identification of the enrichment of the “E2F targets” and “G2/M checkpoint” in the E2-treated MCF7 cells at the proteome levels, suggests that these two molecular processes are likely the primary points at which E2 drives the cell cycle progression in ERα-positive breast cancer cells.

**Figure 2:**
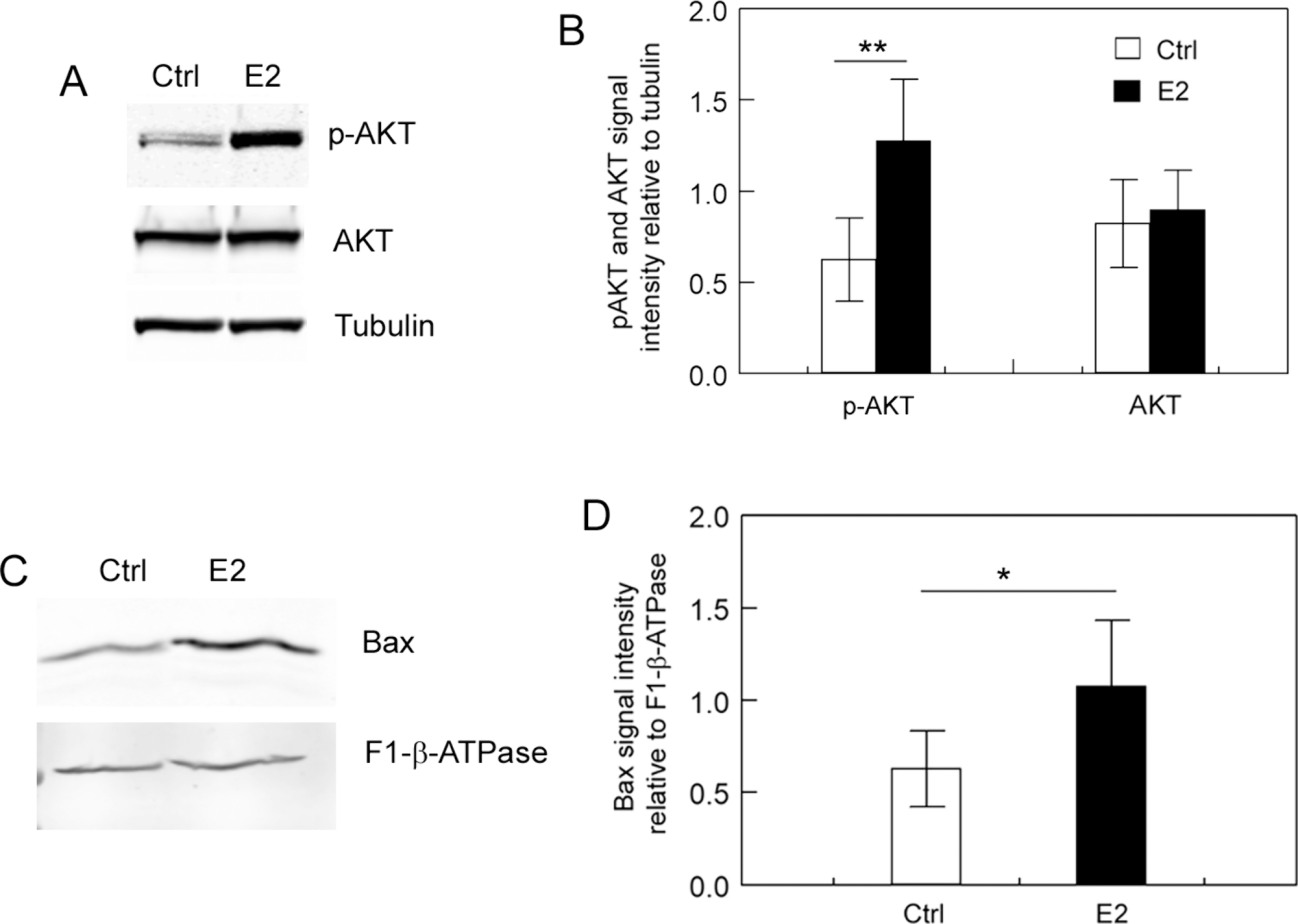
Validation of PI3K signaling activation and the elevation of Bax in the membrane/organelle fraction of E2-treated MCF7 cells. (A) Validation of PI3K signaling activation. MCF7 cells were either mock-treated or treated with 10 nM E2 for 10 min. AKT Ser-473 phosphorylation was analyzed using Western blotting with the indicated antibodies. The experiments were performed in five replicates using independently prepared biological samples (*n* = 5). Panel A shows one of the Western blots, and uncropped images of the five Western blot replicates are shown in Supporting Figure S2. (B) Quantification of p-AKT and AKT signal intensities. Signal intensities were quantified relative to tubulin in Western blots (*n* = 5). As depicted, while the AKT protein level remained unchanged, AKT Ser-473 phosphorylation significantly increased in E2-treated cells compared to mock-treated cells. (C) Validation of the elevation of Bax protein in the membrane/organelle fraction of the E2-treated MCF7 cells. MCF7 cells were either mock-treated or treated with 100 nM E2 for 18 h. Bax protein levels in the membrane/organelle fraction were analyzed using Western blotting with an anti-Bax antibody. The experiments were performed in five replicates using independently prepared biological samples (*n* = 5). Panel C shows one of the Western blots, and uncropped images of the five Western blot replicates are shown in Supporting Figure S3. (D) Quantification of Bax signal intensities. Bax signal intensities were quantified relative to F1-β-ATPase in Western blots (*n* = 5). Tubulin and the mitochondrion inner membrane protein F1-β-ATPase served as loading controls for the whole-cell lysate and membrane/organelle proteins, respectively. *p < 0.05; **p < 0.01.

Consistent with the report that ERα interacts with the p85α regulatory subunit of PI3K,^7^ the GSEA analysis revealed the enrichment of the proteins that regulate the PI3K signaling following E2 treatment (Table 2). To confirm the predicted changes in the PI3K signaling in the E2-treated cells relative to the control cells, we assessed the phosphorylation and activation of AKT/PKB, a key downstream kinase of PI3K, in E2-treated MCF7 cells using Western blotting. Our Western blot analysis results (Figure 2A-B) were consistent with the GSEA prediction, confirming the activation of PI3K signaling by E2 in MCF7 cells. The phosphorylation and activation of AKT/PKB by E2 in MCF7 cells has also been shown by others.^45^ The PI3K/AKT/mTOR signaling is a key regulator of cell growth.^46^ The activated PI3K/AKT/mTOR signaling may contribute to the expression of the E2F target genes,^47^ along with other factors such as upregulation of cyclin D1 and activation of CDK4/6, which are known to be associated with the induction of E2F-mediated transcription.^48^ The E2-regulated cytosolic, nuclear, and membrane/organelle proteins were also analyzed using the PID gene sets in GSEA.^33^ Most PID gene sets that were overrepresented in the E2-treated cells relative to the control cells supported cell division (Table 2). Thus, our proteomic data support the notion that one of the primary effects of E2 in ERα-positive breast cancer cells is to promote cell division.

### The Expression of the Proteins Involved in Nuclear Import, Protein Translation, and mRNA/Protein Stability Was Enriched in the E2-Treated Cells

Among the 77 cytosolic proteins, 74 nuclear proteins, and 81 membrane/organelle proteins significantly regulated by E2 (Figure 1A–C; Supporting Tables S4–S6), 24 proteins were quantified in two of the three fractions (bold in Supporting Tables S4–S6). Of the 24 proteins, 20 were consistently upregulated or downregulated by E2 in both fractions, and 4 exhibited upregulation in one fraction but downregulation in another. We pooled the differentially expressed cytosolic, nuclear, and membrane/organelle proteins for further bioinformatics analysis. During the pooling, we included the 20 proteins that changed in the same direction and excluded the 4 proteins that changed in the opposite direction. The data pooling resulted in a list of 204 E2-regulated proteins (Supporting Table S7). Among these 204 proteins, E2 upregulated 124 and downregulated 80 proteins. Thus, the number of E2-upregulated proteins was 55% higher than that of E2-downregulated proteins, suggesting that E2 influences MCF7 cells primarily through protein upregulation rather than downregulation.

To understand how the E2-regulated proteins influence breast cancer cell division and apoptosis, we analyzed the E2-upregulated and E2-downregulated proteins with DAVID, a web server for functional annotation and enrichment analyses of gene/protein lists.^34^ In this analysis, we first determined the sets of genes/proteins overrepresented in the E2-upregulated and E2-downregulated proteins by performing Gene Ontology (GO) enrichment analysis. Cluster analysis of the E2-upregulated proteins using the GO biological processes (BP) (GOTERM_BP_DIRECT) and GO molecular functions (MF) (GOTERM_MF_DIRECT) revealed that the proteins involved in protein nuclear import, protein translation, mRNA stability, protein folding/stability, and nucleosome assembly were enriched in the E2-upregulated proteins (Table 3). All of these changes appear to facilitate cell division. To strengthen this notion, we further analyzed the E2-upregulated proteins with FunRich, a stand-alone software tool for functional enrichment and interaction network analysis of genes and proteins.^35^ Consistent with the results from the DAVID analysis, when the E2-upregulated proteins were analyzed with FunRich for biological pathways, the proteins involved in gene expression, protein translation, and mRNA splicing were significantly enriched in the E2-upregulated proteins (Figure 1D). Thus, it is likely that E2 may promote MCF7 cell division (Table 2) by enhancing various molecular processes that are supportive of cell division, including protein nuclear import, protein translation, mRNA stability, protein folding, and nucleosome assembly (Table 3; Figure 1D). Interestingly, when the E2-upregulated proteins were analyzed with FunRich to determine the enrichment of cellular components, proteins related to the centrosome were enriched (Figure 1E). Centrosomes are prominent during the M phase when the two daughter cells are forming, which happens in the last hour of the cell cycle.^49^ The enrichment of centrosome proteins in the E2-upregulated proteins suggests that the E2-treated cells are more active in cell division compared to vehicle-treated cells. This observation is consistent with the results from the functional annotation analysis (Table 3; Figure 1D) and the GSEA analysis (Table 2).

Different from the E2-upregulated proteins, only the actin/actin filament binding and actin/actin filament-based motor activities were enriched in the E2-downregulated proteins (Supporting Table S8). Actin and actin filaments play key roles in both the division of the nucleus (karyokinesis) and the division of the cytoplasm (cytokinesis). The cellular actin network undergoes dynamic changes during cell division, including vigorous disassembly and reassembly of actin filaments.^50^ Whether E2 can promote cell division by inhibiting actin/actin filament binding and actin/actin filament-based motor activity warrants further investigation.

### Many E2-Upregulated Proteins Contain the HEAT, KH, and RRM Domains

We also determined the sequence features of E2-regulated proteins through Functional annotation analysis using UP_SEQ_FEATURES in DAVID. Interestingly, many of the E2-upregulated proteins contained the HEAT (Huntingtin, elongation factor 3, protein phosphatase 2A, and TOR1), KH (K homology) domains, and RRM (RNA recognition motif) (Table 4). The HEAT repeat is a structural motif found in proteins and was named after the four proteins in which it was initially identified: Huntingtin, elongation factor 3 (EF3), protein phosphatase 2A (PP2A), and TOR1. The HEAT domain is characterized by a tandem repeat of two α-helices connected by a short loop. These repeats can vary in number, ranging from 3 to 30 in a single protein. HEAT domain-containing proteins participate in various cellular processes, including protein–protein interactions, cellular organization, and cell cycle regulation. Proteins with HEAT domains are often associated with cellular growth and proliferation.^51^ The enrichment of HEAT domains in the E2-upregulated proteins suggests that, apart from breast cancer, estrogen may also be related to other diseases, such as neurodegenerative conditions like Huntington’s Disease and Alzheimer’s Disease.^52^

The KH (K homology) domain is a protein domain that is named after the first protein in which it was identified, the human heterogeneous nuclear ribonucleoprotein K (hnRNP K). The KH domain is a small, highly conserved RNA-binding domain found in various proteins involved in RNA metabolism and regulation. The KH domain is approximately 70 amino acids long and adopts a compact, globular structure. It is characterized by a three-stranded β-sheet and an α-helix, forming a characteristic fold.^53^ The domain contains two conserved motifs, the GxxG, and the KH box, contributing to RNA binding. The GxxG motif stabilizes the RNA-binding site, while the KH box interacts directly with the RNA molecule.^53^ KH domains are known to bind single-stranded RNA, and their specificity and affinity for RNA can vary depending on the protein context. Proteins containing KH domains are involved in various RNA-related processes, including RNA splicing, mRNA stabilization and localization, translation regulation, and microRNA processing.^53^

The RNA recognition motif (RRM), also known as the RNA-binding domain (RBD), is a common and versatile protein domain in many proteins involved in RNA binding and processing. RRMs are approximately 90 amino acids long and are characterized by a conserved RNA-binding fold. This fold consists of four antiparallel beta strands and two alpha helices, forming a compact structure with a characteristic beta-alpha-beta–beta-alpha-beta topology.^54^ The RRM domain typically contains conserved aromatic residues involved in RNA recognition. RRM domains are crucial in various RNA-related processes, including pre-mRNA splicing, mRNA stability and localization, translation regulation, and RNA processing.^54^ Proteins containing RRM domains can recognize specific RNA sequences or structures and interact with RNA molecules to mediate their function. Many RNA-binding proteins, such as heterogeneous nuclear ribonucleoproteins (hnRNPs), splicing factors, RNA helicases, and poly(A)-binding proteins, contain one or multiple RRM domains.^54^ The versatility of the RRM domain allows it to interact with RNA in a modular and specific manner, enabling it to participate in diverse RNA-related processes within the cell.

The observation that many E2-upregulated proteins contain the HEAT, KH, and RRM domains is consistent with the results from the DAVID analysis, which suggested that E2 may promote MCF7 cell proliferation mainly by affecting the expression of the proteins involved in protein nuclear import, protein translation, protein folding, and mRNA stability (Table 3; Figure 1D). In contrast, among the E2-downregulated proteins, only the RRM domains showed moderate enrichment, and no other domains displayed significant enrichment (Supporting Table S9).

### E2 Regulates the Abundance of the Proteins That Promote or Inhibit Apoptosis in MCF7 Cells

In addition to affecting cell division, the DAVID analysis revealed that the proteins involved in cytochrome c release were enriched in the E2-upregulated proteins (Table 3). Cytochrome c release from mitochondria is a critical step in mitochondrion-dependent apoptosis. Consistent with this, our proteomic data revealed that the levels of two pro-apoptotic proteins, Bax and Bid, in the membrane/organelle fraction were significantly higher in E2-treated cells compared to vehicle-treated cells (Supporting Table S6). Bax is known to translocate from the cytosol to the mitochondria to initiate mitochondrion-dependent apoptosis by inducing cytochrome c release from the intermembrane space of mitochondria.^55−57^ We validated the higher level of Bax protein in the membrane/organelle fraction in the E2-treated MCF7 cells relative to vehicle-treated cells by performing Western blot analysis (Figure 2C–D). Thus, it is likely that the changes in the abundance of the proteins related to cytochrome c release in the E2-treated cells relative to the control cells are linked to the elevated levels of the pore-forming protein Bax in the mitochondria. These results suggest that induction of mitochondrion-dependent apoptosis is potentially an important physiological function of E2. Although E2 has been reported to induce apoptosis through the death receptor-activated apoptotic pathway,^6,58^ we did not observe this at the proteome level. This discrepancy could potentially be due to the fact that death receptor-activated cell death has been observed in cells that are typically long-depleted of estrogen, such as triple-negative breast cancer cells or in patients undergoing antiestrogen therapy.^58,59^ Contrary to the effect of E2 on the abundance of the proteins related to cytochrome c release (Table 3), the GSEA analysis revealed that the proteins involved in enhancing the PI3K/AKT/mTOR signaling pathway, a major apoptosis-inhibitory pathway in mammalian cells,^60^ were enriched in the E2-treated cells compared to the control cells (Table 2). It is known that the activated PI3K leads to activation of AKT, which in turn inactivates the proapoptotic protein Bad and other factors.^61^ Inhibition of PI3K leads to cell apoptosis.^62,63^ Thus, our proteomic and subsequent bioinformatics analyses suggest that E2 potentially has a dual effect on apoptosis in MCF7 cells. It may promote apoptosis through affecting the abundance of proteins related to cytochrome c release(Table 3) and simultaneously inhibit apoptosis by regulating the PI3K signaling (Table 2). We verified the predicted activation of PI3K in E2-treated MCF7 cells by determining the phosphorylation of the key PI3K downstream kinase, AKT/PKB, using Western blotting (Figure 2A-B). Additionally, we indirectly assessed cytochrome c release from the mitochondria in the cells by determining the levels of the pore-forming protein Bax in the membrane/organelle fraction, also using Western blotting. The Western blot results (Figure 2C–D) were consistent with the proteomic results (Supporting Table S6). Our results align with previous molecular and cellular studies that demonstrate the complex effects of estrogen on apoptosis, which can be both stimulatory and inhibitory.^6,58^

**Table 3:** Functional Annotation Analysis by DAVID of the Proteins Whose Expression Was Significantly Upregulated by E2: with GOTERM_BP_DIRECT and GOTERM_MF_DIRECT^a^

category	term	count	UniProt ID	*p* value	fold enrichment
cluster 1	enrichment score: 4.78				
GOTERM_MF_DIRECT	GO:0031267 ∼ small GTPase binding	13	Q14974, O00429, O14980, Q07960, Q8TEX9, Q9Y3P9, Q96P70, O95373, O60610, Q92973, Q13492, O14787, P55060	3.76 × 10^–07^	6.9
GOTERM_MF_DIRECT	GO:0061608 ∼ nuclear import signal receptor activity	5	Q14974, Q92973, O14787, Q8TEX9, Q96P70	7.09 × 10^–06^	38.8
GOTERM_BP_DIRECT	GO:0006606 ∼ protein import into nucleus	7	Q14974, Q92973, O14787, P55060, Q8TEX9, Q96P70, O95373	4.17 × 10^–05^	11.0
GOTERM_MF_DIRECT	GO:0008139 ∼ nuclear localization sequence binding	4	Q14974, Q92973, O14787, Q8TEX9	6.65 × 10^–04^	23.0
cluster 2	enrichment score: 3.66				
GOTERM_BP_DIRECT	GO:0006413 ∼ translational initiation	7	O15371, Q04637, Q99613, O75821, O00571, Q15056, P60842	9.59 × 10^–07^	20.9
GOTERM_MF_DIRECT	GO:0003743 ∼ translation initiation factor activity	6	O15371, Q04637, Q99613, O75821, Q15056, P60842	5.52 × 10^–05^	14.6
GOTERM_BP_DIRECT	GO:0001732 ∼ formation of cytoplasmic translation initiation complex	3	O15371, Q99613, O75821	0.0046	29.0
GOTERM_MF_DIRECT	GO:0008135 ∼ translation factor activity, RNA binding	3	Q04637, Q15056, P60842	0.0094	20.2
cluster 3	enrichment score: 2.69				
GOTERM_BP_DIRECT	GO:1900152 ∼ negative regulation of nuclear-transcribed mRNA catabolic process, deadenylation-dependent decay	3	P67809, P11940, O60506	0.0013	54.8
GOTERM_BP_DIRECT	GO:0070934 ∼ CRD-mediated mRNA stabilization	3	P67809, P11940, O60506	0.0019	44.9
GOTERM_BP_DIRECT	GO:2000767 ∼ positive regulation of cytoplasmic translation	3	P67809, P11940, O60506	0.0036	32.9
cluster 4	enrichment score: 2.67				
GOTERM_BP_DIRECT	GO:0061077 ∼ chaperone-mediated protein folding	6	P50990, O75190, P78371, P49368, P04792, Q02790	3.57 × 10^–06^	25.3
GOTERM_MF_DIRECT	GO:0051082 ∼ unfolded protein binding	7	P50990, O00170, O75190, Q8N163, P78371, P49368, P04792	1.88 × 10^–04^	8.4
GOTERM_MF_DIRECT	GO:0044183 ∼ protein binding involved in protein folding	5	P50990, O75190, P78371, P49368, P04792	3.14 × 10^–04^	15.2
GOTERM_BP_DIRECT	GO:0032212 ∼ positive regulation of telomere maintenance via telomerase	4	P50990, P78371, P49368, P09651	0.0011	19.4
GOTERM_BP_DIRECT	GO:1904851 ∼ positive regulation of establishment of protein localization to telomere	3	P50990, P78371, P49368	0.0016	49.4
GOTERM_BP_DIRECT	GO:1904871 ∼ positive regulation of protein localization to Cajal body	3	P50990, P78371, P49368	0.0019	44.9
GOTERM_BP_DIRECT	GO:1904874 ∼ positive regulation of telomerase RNA localization to Cajal body	3	P50990, P78371, P49368	0.0036	32.9
GOTERM_BP_DIRECT	GO:0050821 ∼ protein stabilization	6	P50990, P78371, P46379, P49368, P83436, O60716	0.0122	4.3
GOTERM_MF_DIRECT	GO:0016887 ∼ ATPase activity	8	P50990, P78371, P33993, O00571, P49368, P49736, O00148, P60842	0.0181	3.0
GOTERM_BP_DIRECT	GO:0007339 ∼ binding of sperm to zona pellucida	3	P50990, P78371, P49368	0.0242	12.3
GOTERM_BP_DIRECT	GO:0006457 ∼ protein folding	5	P50990, O75190, P78371, P49368, Q02790	0.0253	4.5
GOTERM_BP_DIRECT	GO:1901998 ∼ toxin transport	3	P50990, P78371, P49368	0.0265	11.8
cluster 5	enrichment score: 1.75				
GOTERM_BP_DIRECT	GO:0001836 ∼ release of cytochrome c from mitochondria	3	O00429, Q07812, P55957	0.0084	21.5
GOTERM_BP_DIRECT	GO:0090200 ∼ positive regulation of release of cytochrome c from mitochondria	3	O00429, Q07812, P55957	0.0114	18.3
GOTERM_BP_DIRECT	GO:2001244 ∼ positive regulation of intrinsic apoptotic signaling pathway	3	O00429, Q07812, P55957	0.0209	13.3
GOTERM_BP_DIRECT	GO:0043065 ∼ positive regulation of apoptotic process	6	O00429, P50570, Q8N163, Q07812, O00571, P55957	0.0480	3.0
cluster 6	enrichment score: 1.58				
GOTERM_BP_DIRECT	GO:0006334 ∼ nucleosome assembly	5	P49321, Q01105, P16403, P49736, P16402	0.0144	5.4
GOTERM_MF_DIRECT	GO:0042393 ∼ histone binding	5	P49321, Q01105, Q96P70, P49736, O95373	0.0342	4.1
GOTERM_BP_DIRECT	GO:0006260 ∼ DNA replication	4	P49321, Q01105, P33993, P49736	0.0368	5.4
cluster 7	enrichment score: 1.19				
GOTERM_BP_DIRECT	GO:0071902 ∼ positive regulation of protein serine/threonine kinase activity	3	P36507, O00571, Q02750	0.0265	11.8

a Only the categories and terms with *p* < 0.05 are listed.

**Table 4:** Functional Annotation Analysis by DAVID Software of the Proteins Whose Expression Was Significantly Upregulated by E2: with UP_SEQ_FEATURE^a^

category	term	count	UniProt ID	*p* value	fold enrichment
cluster 1	enrichment score: 6.37				
UP_SEQ_FEATURE	DOMAIN:Importin N-terminal	8	Q14974, O14980, Q92973, O14787, P55060, Q8TEX9, Q96P70, O95373	2.13 × 10^–12^	84.9
UP_SEQ_FEATURE	REPEAT:HEAT 4	9	Q14974, Q6AI08, O14980, Q92973, P53618, Q86VP6, P30153, O14787, Q8TEX9	4.68 × 10^–10^	30.6
UP_SEQ_FEATURE	REPEAT:HEAT 3	9	Q14974, Q6AI08, O14980, Q92973, P53618, Q86VP6, P30153, O14787, Q8TEX9	1.39 × 10^–09^	26.8
UP_SEQ_FEATURE	REPEAT:HEAT 6	8	Q14974, O14980, Q92973, P53618, Q86VP6, P30153, O14787, Q8TEX9	1.73 × 10^–09^	36.7
UP_SEQ_FEATURE	REPEAT:HEAT 1	9	Q14974, Q6AI08, O14980, Q92973, P53618, Q86VP6, P30153, O14787, Q8TEX9	4.63 × 10^–09^	23.2
UP_SEQ_FEATURE	REPEAT:HEAT 2	9	Q14974, Q6AI08, O14980, Q92973, P53618, Q86VP6, P30153, O14787, Q8TEX9	4.63 × 10^–09^	23.2
UP_SEQ_FEATURE	REPEAT:HEAT 5	8	Q14974, O14980, Q92973, P53618, Q86VP6, P30153, O14787, Q8TEX9	5.27 × 10^–09^	31.6
UP_SEQ_FEATURE	REPEAT:HEAT 10	6	Q14974, O14980, Q92973, Q86VP6, P30153, O14787	2.43 × 10^–07^	42.4
UP_SEQ_FEATURE	REPEAT:HEAT 9	6	Q14974, O14980, Q92973, Q86VP6, P30153, O14787	3.03 × 10^–07^	40.8
UP_SEQ_FEATURE	REPEAT:HEAT 8	6	Q14974, O14980, Q92973, Q86VP6, P30153, O14787	5.52 × 10^–07^	36.4
UP_SEQ_FEATURE	REPEAT:HEAT 7	6	Q14974, O14980, Q92973, Q86VP6, P30153, O14787	1.11 × 10^–06^	31.8
UP_SEQ_FEATURE	REPEAT:HEAT 15	5	Q14974, Q92973, Q86VP6, P30153, O14787	1.44 × 10^–06^	56.6
UP_SEQ_FEATURE	REPEAT:HEAT 14	5	Q14974, Q92973, Q86VP6, P30153, O14787	2.48 × 10^–06^	49.9
UP_SEQ_FEATURE	REPEAT:HEAT 12	5	Q14974, Q92973, Q86VP6, P30153, O14787	3.18 × 10^–06^	47.2
UP_SEQ_FEATURE	REPEAT:HEAT 13	5	Q14974, Q92973, Q86VP6, P30153, O14787	3.18 × 10^–06^	47.2
UP_SEQ_FEATURE	REPEAT:HEAT 11	5	Q14974, Q92973, Q86VP6, P30153, O14787	4.99 × 10^–06^	42.4
UP_SEQ_FEATURE	REPEAT:HEAT 17	4	Q14974, Q92973, Q86VP6, O14787	2.26 × 10^–05^	67.9
UP_SEQ_FEATURE	REPEAT:HEAT 18	4	Q14974, Q92973, Q86VP6, O14787	2.26 × 10^–05^	67.9
UP_SEQ_FEATURE	REPEAT:HEAT 19	4	Q14974, Q92973, Q86VP6, O14787	2.26 × 10^–05^	67.9
UP_SEQ_FEATURE	REPEAT:HEAT 16	4	Q14974, Q92973, Q86VP6, O14787	3.10 × 10^–05^	61.7
UP_SEQ_FEATURE	REPEAT:HEAT 20	3	Q92973, Q86VP6, O14787	0.0012	56.6
UP_SEQ_FEATURE	REPEAT:HEAT	3	Q14974, P30153, Q8TEX9	0.0072	23.1
cluster 2	enrichment score: 5.97				
UP_SEQ_FEATURE	DOMAIN:KH 1	6	Q96AE4, Q96I24, P61978, Q15366, P51114, Q15365	2.43 × 10^–07^	42.4
UP_SEQ_FEATURE	DOMAIN:KH 2	6	Q96AE4, Q96I24, P61978, Q15366, P51114, Q15365	2.43 × 10^–07^	42.4
UP_SEQ_FEATURE	DOMAIN:KH 3	5	Q96AE4, Q96I24, P61978, Q15366, Q15365	1.06 × 10^–06^	60.6
UP_SEQ_FEATURE	DOMAIN:K Homology	5	Q96AE4, Q96I24, P61978, Q15366, Q15365	2.03 × 10^–05^	30.3
cluster 3	enrichment score: 4.87				
UP_SEQ_FEATURE	DOMAIN:RRM	11	P52597, P31942, Q13148, O75821, P98179, P11940, Q8N684, P09651, O60506, P26599, Q15056	1.43 × 10^–06^	7.8
UP_SEQ_FEATURE	DOMAIN:RRM 1	8	P52597, P31942, Q13148, Q13151, P11940, P09651, O60506, P26599	2.76 × 10^–06^	12.9
UP_SEQ_FEATURE	DOMAIN:RRM 2	8	P52597, P31942, Q13148, Q13151, P11940, P09651, O60506, P26599	2.76 × 10^–06^	12.9
UP_SEQ_FEATURE	DOMAIN:RRM 3	4	P52597, P11940, O60506, P26599	0.0031	13.6
cluster 4	enrichment score: 1.29				
UP_SEQ_FEATURE	DOMAIN:DEAD-box RNA helicase Q	3	O00571, O00148, P60842	0.0132	17.0
UP_SEQ_FEATURE	MOTIF:Q motif	3	O00571, O00148, P60842	0.0228	12.7

a Only the categories and terms with *p* < 0.05 are listed.

### A Combination of E2 and a PI3K Inhibitor Significantly Increases Apoptosis in MCF7 Cells

**Figure 3:**
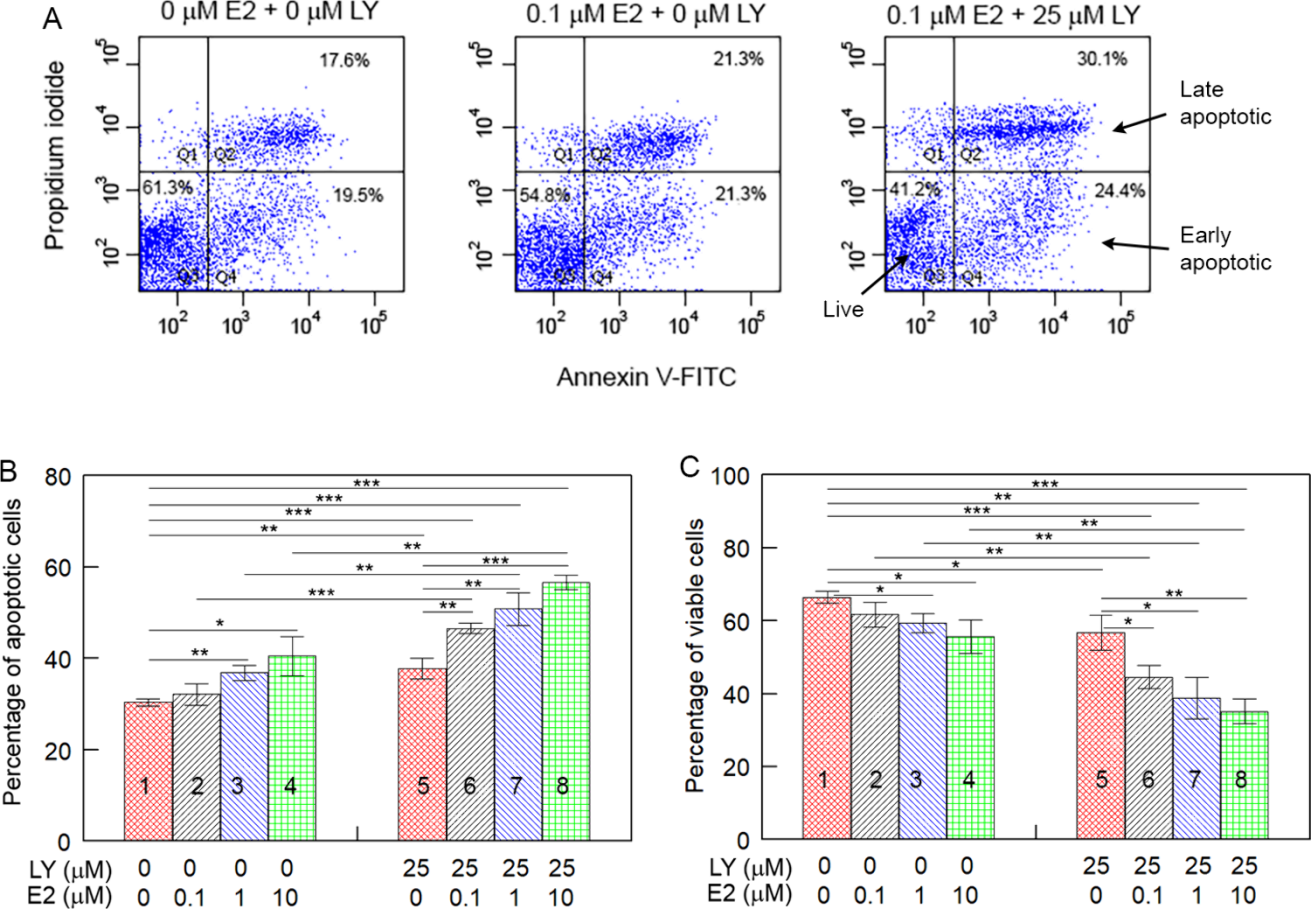
Inhibition of the PI3K enhances the stimulatory effect of E2 on apoptosis in MCF7 cells. (A) Representative flowcharts showing the effects of E2 (compare the middle chart to the left chart) or a combination of E2 and the PI3K inhibitor Ly294002 (compare the right chart to the middle and left charts) on apoptosis in MCF cells. (B and C) The effects of E2 or a combination of E2 and the PI3K inhibitor Ly294002 on apoptosis (sum of early and late apoptosis; B) and viability (C) in MCF cells. MCF7 cells were treated with indicated concentrations of E2 in the presence or absence of the PI3K inhibitor Ly294002. The cells were then stained with annexin V-FITC and propidium iodide, and apoptosis was assessed using a flow cytometer. Values in B and C are the mean ± SD of three separate sample preparations. LY: Ly294002. *p < 0.05; **p < 0.01; ***p < 0.001.

Since E2 may promote and inhibit apoptosis simultaneously (Tables 1 and 2),^6,58^ we hypothesized that blocking the inhibitory effect of E2 could significantly enhance its stimulatory effect on apoptosis. To assess this possibility, we treated MCF7 cells with varying concentrations of E2 in the presence or absence of the PI3K inhibitor Ly294002. We then stained the cells with annexin V and propidium iodide and assessed apoptosis using a flow cytometer (Figure 3A). In the absence of Ly294002, while apoptosis in cells treated with 1 μM and 10 μM E2 were 21.4% (p = 0.0034) and 31.6% (p = 0.016) higher than in vehicle-treated cells, respectively (Figure 3B, compare columns 3 and 4 with 1), 0.1 μM E2 did not induce significant apoptosis when compared to the vehicle control (compare column 2 with 1). In the presence of Ly294002, apoptosis in cells treated with 0.1 μM, 1 μM, and 10 μM E2 were 23.4% (p = 0.0000), 34.7% (p = 0.0006), and 50.0% (p = 0.0000) higher than in vehicle-treated cells, respectively, with all differences being statistically significant (compare columns 6, 7, and 8 with 1). Furthermore, apoptosis in cells treated with both E2 and Ly294002 was significantly higher than in cells treated with E2 alone at all three E2 concentrations used (compare columns 6 with 2, columns 7 with 3, and columns 8 with 4) (p = 0.0007, 0.0036, and 0.0037, respectively). Additionally, apoptosis in cells treated with E2 and Ly294002 was also significantly higher than in cells treated with Ly294002 alone (p = 0.0039, 0.0060, 0.0003, respectively; compare columns 6, 7, and 8 with 5). The effect of E2 on the percentage of viable cells in the presence of Ly294002 compared to its absence was consistent with the results on apoptosis (Figure 3C). These results demonstrate that by blocking the PI3K pathway, the effect of E2 on apoptosis in MCF7 cells can be substantially enhanced.

## Discussion

In this study, we used a stable isotope labeling-based quantitative proteomics method coupled with subcellular fractionation to comprehensively assess the impact of E2 on protein expression in MCF7 cells. Our findings suggest that E2 may promote MCF7 cell division primarily by inducing the expression of E2F target genes and enhancing the G2/M checkpoint transition (Table 2). The stimulatory effect of estrogen on the G1 to S phase transition has been reported.^64,65^ Therefore, it appears that one key mechanism by which estrogen drives cell division in ERα-positive cells is by promoting progression through the restriction point. While the effect of estrogen on the G2/M checkpoint has been reported,^44^ its physiological impact remains elusive. Notably, the G2/M checkpoint is a DNA damage checkpoint, and unchecked progression at this point may increase the risk of mutations in the cell. Additional investigation into the stimulatory effect of estrogen on the G2/M checkpoint may be warranted, as the results could provide insights into the potential association between estrogen’s influence at this stage and its pathogenic effects.

Our proteomic and bioinformatics analyses suggest a complex role of E2 in apoptosis in MCF7 cells. On the one hand, the abundance of the proteins involved in cytochrome c release from the mitochondria, including the levels of the pore-forming protein Bax in the membrane/organelle fraction, was altered by E2 (Table 3, Figure 2C–D, and Supporting Table S6). Cytochrome c release is a crucial event in the mitochondrion-dependent apoptotic pathway. On the other hand, E2 treatment led to the enrichment of the proteins related to the PI3K/AKT/mTOR pathway among the E2-regulated proteins (Table 2). E2 has been shown to regulate PI3K through the binding of ERα with the p85α regulatory subunit of PI3K.^7^ We assessed the PI3K signaling in the E2- and vehicle-treated MCF7 cells by determining the phosphorylation of the key PI3K downstream kinase AKT/PKB using Western blotting. Our Western blot results (Figure 2A-B) were consistent with the proteomic and bioinformatics prediction (Table 2). The phosphorylation and activation of AKT/PKB in MCF7 have also been reported by others.^45^ Since the PI3K pathway is a major antiapoptotic pathway in mammalian cells,^60^ it is evident that the antiapoptotic effect of E2 is also discernible at the proteome level. Therefore, in addition to its mitogenic property of stimulating ERα-positive breast cancer cell division, E2 can also fundamentally influence apoptosis. Given its dual role in enhancing and inhibiting apoptosis (Tables 2 and 3),^6^ we conducted experiments to determine whether blocking the apoptosis-inhibitory PI3K could enhance the stimulatory effect of E2 on apoptosis in MCF7 cells. Our results demonstrate that apoptosis in MCF7 cells treated with a combination of E2 and the PI3K inhibitor Ly294002 was significantly higher than that in cells treated with E2 alone, suggesting that by blocking the E2-induced PI3K pathway, the effect of E2 on apoptosis in MCF7 cells can be substantially enhanced. It might be worthwhile to investigate further the effects of a combination of E2 and a PI3K inhibitor on apoptosis *in vivo* in the future, as the results could shed light on the potential application of this strategy in treating ERα-positive cancer. Furthermore, several practical issues must be addressed in future studies. For instance, what is the lowest concentration of E2 that is effective in inducing apoptosis when E2 and a PI3K inhibitor are used in the treatment? If the lowest required concentration is higher than the typical pharmacological concentrations of E2 achieved by conventional means, is it possible to increase the local E2 concentration in tumor cells?

The PI3K pathway may play a pivotal role in mediating the physiological effects of estrogen. Once activated by estrogen, presumably via the binding of ERα to the p85 subunit of PI3K,^7^ the activated PI3K catalyzes the synthesis of phosphatidylinositol-3,4,5-trisphosphate (PIP3), resulting in the phosphorylation of AKT/PKB. On the one hand, the phosphorylated and activated AKT/PKB mediates the stimulatory effect of estrogen on breast cancer cells by inhibiting GSK3β, leading to the activation of the Wnt-β-catenin pathway.^66^ In line with this expected effect, our GSEA analysis revealed that the abundance of the proteins related to the Wnt-β-catenin pathway was enriched in E2-treated MCF7 cells compared to vehicle-treated control cells (Table 2). Thus, in addition to other molecular processes that are stimulated by estrogen during the cell cycle, for example, upregulation of cyclins A, B, D1, and E, and cyclin-dependent kinases (CDKs),^64^ and the interaction of activated ERα with cyclins, CDKs, CDK inhibitors, and the retinoblastoma protein,^67^ the PI3K signaling appears to contribute significantly to the mitogenic effect of estrogen in breast cancer cells. On the other hand, the activated AKT/PKB can also inhibit apoptosis by inactivating the proapoptotic protein Bad.^61^ In this study, we demonstrated that E2 treatment led to the enrichment of the proteins involved in the PI3K signaling among the E2-regulated proteins (Table 2) and to the phosphorylation of AKT/PKB (Figure 2A-B), a finding also reported by others.^45^ Therefore, the estrogen-activated PI3K signaling pathway likely plays a major role in mediating the effects of estrogen on both cell division and apoptosis. Inhibition of PI3K activity, on the one hand, can suppress the estrogen-induced G1 to S phase transition in the cell cycle, mimicking the effect of CDK inhibitors.^68,69^ On the other hand, it can enhance estrogen’s apoptosis-inducing effect in ERα-positive breast cancer cells.

Another cellular factor that may play a pivotal role in mediating the physiological effects of estrogen in ERα-positive breast cancer cells is the proto-oncogene c-Myc. c-Myc is a transcription factor involved in various cellular processes, including cell growth, proliferation, metabolism, differentiation, stress responses, apoptosis, and drug resistance.^70^ Dysregulation of c-Myc is implicated in the development of multiple types of cancer, including breast cancer.^70^ c-Myc is a target gene of ERα.^71^ Consistent with this, our GSEA analysis showed that the proteins involved in c-Myc were enriched among the E2-regulated proteins (Table 2). If this holds true, the estrogen-activated c-Myc can lead to downstream pathway activation, including the G1 to S phase transition in the cell cycle,^64^ which was a significant molecular event predicted to be influenced by E2 in MCF7 cells in this study (Table 2). Therefore, in addition to the PI3K/AKT/mTOR pathway, c-Myc may also play a pivotal role in mediating the mitogenic effect of estrogen in breast cancer cell division.

Furthermore, c-Myc has also been shown to regulate apoptosis. For instance, c-Myc can induce apoptosis by upregulating the expression of pro-apoptotic genes including Bax.^72,73^ Upregulated c-Myc can also disrupt mitochondrial membrane potential, promoting mitochondrion-dependent apoptosis.^74^ Consistent with these expected effects, our proteomic data revealed that the protein levels of Bax in the membrane/organelle fraction of the E2-treated cells were significantly elevated compared to vehicle-treated cells (Supporting Table S6); the subsequent bioinformatics analysis revealed that the proteins involved in cytochrome c release from mitochondria was enriched among the E2-upregulated proteins in MCF7 cells (Table 3). It is noticeable that Bax protein levels in E2-treated cells were higher than their counterparts in the membrane/organelle fraction (Supporting Table S6), but not in the cytosolic and nuclear fractions (Supporting Tables 4 and 5). It is well established that the Bax protein migrates from the cytosol to the mitochondria upon apoptosis induction.^55−57^ While the potential role of c-Myc in upregulating Bax expression is known,^72,73^ it is more likely that the higher levels of Bax in the membrane/organelle fraction of the E2-treated MCF7 cells result from the translocation of Bax from the cytosol to the mitochondria. Regardless of the underlying mechanism for the higher levels of Bax in the membrane/organelle fraction of E2-treated cells, it is expected that the higher levels of the pore-forming protein Bax could significantly contribute to the mitochondrion-dependent apoptosis. In summary, PI3K and c-Myc likely play pivotal roles in mediating the physiological effects of estrogen, including its impact on cell division and apoptosis, in ERα-positive breast cancer cells.

## Data Availability

The MS proteomic data have been deposited in the ProteomeXchange Consortium (http://proteomecentral.proteomexchange.org) via the PRoteomics IDEntifications (PRIDE) partner repository with the data set identifier PXD049343.

## Supporting Information Available

Supporting Figures: Supporting Figure S1, fractionation of cellular proteins into cytosolic, nuclear, and membrane/organelle fractions. Supporting Figure S2, uncropped images of the Western blots showing AKT Ser-473 phosphorylation in E2-treated MCF7 cells (E2) compared to vehicle-treated control cells (Ctrl). Supporting Figure S3, uncropped images of the Western blots showing elevated Bax protein levels in the membrane/organelle fraction of E2-treated MCF7 cells (E2) compared to vehicle-treated control cells (Ctrl) (PDF)
Supporting Table S1, list of quantified cytosolic proteins (XLSX)
Supporting Table S2, list of quantified nuclear proteins (XLSX)
Supporting Table S3, list of quantified membrane/organelle proteins (XLSX)
Supporting Table S4, list of E2-regulated cytosolic proteins (XLSX)
Supporting Table S5, list of E2-regulated nuclear proteins (XLSX)
Supporting Table S6, list of E2-regulated membrane/organelle proteins (XLSX)
Supporting Table S7, list of E2-regulated cytosolic, nuclear, and membrane/organelle proteins (XLSX)
Supporting Table S8, functional annotation analysis by DAVID of the proteins whose expression was significantly downregulated by E2: with GOTERM_BP_DIRECT and GOTERM_MF_DIRECT (PDF)
Supporting Table S9, functional annotation analysis by DAVID of the proteins whose expression was significantly downregulated by E2: with UP_SEQ_FEATURE (PDF)

## References

[ref1] Chimento A.; De Luca A.; Avena P.; De Amicis F.; Casaburi I.; Sirianni R.; Pezzi V. Estrogen Receptors-Mediated Apoptosis in Hormone-Dependent Cancers. Int. J. Mol. Sci. 2022, 23 (3), 1242 10.3390/ijms23031242. 35163166 PMC8835409

[ref2] JavanMoghadam S.; Weihua Z.; Hunt K. K.; Keyomarsi K. Estrogen receptor alpha is cell cycle-regulated and regulates the cell cycle in a ligand-dependent fashion. Cell Cycle 2016, 15 (12), 1579–90. 10.1080/15384101.2016.1166327. 27049344 PMC4934046

[ref3] Bjornstrom L.; Sjoberg M. Mechanisms of estrogen receptor signaling: convergence of genomic and nongenomic actions on target genes. Mol. Endocrinol. 2005, 19 (4), 833–42. 10.1210/me.2004-0486. 15695368

[ref4] Cheung E.; Acevedo M. L.; Cole P. A.; Kraus W. L. Altered pharmacology and distinct coactivator usage for estrogen receptor-dependent transcription through activating protein-1. Proc. Natl. Acad. Sci. U. S. A. 2005, 102 (3), 559–64. 10.1073/pnas.0407113102. 15642950 PMC545529

[ref5] Bi M.; Zhang Z.; Jiang Y. Z.; Xue P.; Wang H.; Lai Z.; Fu X.; De Angelis C.; Gong Y.; Gao Z.; Ruan J.; Jin V. X.; Marangoni E.; Montaudon E.; Glass C. K.; Li W.; Huang T. H.; Shao Z. M.; Schiff R.; Chen L.; Liu Z. Enhancer reprogramming driven by high-order assemblies of transcription factors promotes phenotypic plasticity and breast cancer endocrine resistance. Nat. Cell Biol. 2020, 22 (6), 701–715. 10.1038/s41556-020-0514-z. 32424275 PMC7737911

[ref6] Lewis-Wambi J. S.; Jordan V. C. Estrogen regulation of apoptosis: how can one hormone stimulate and inhibit?. Breast Cancer Research: BCR 2009, 11 (3), 206 10.1186/bcr2255. 19519952 PMC2716493

[ref7] Simoncini T.; Hafezi-Moghadam A.; Brazil D. P.; Ley K.; Chin W. W.; Liao J. K. Interaction of oestrogen receptor with the regulatory subunit of phosphatidylinositol-3-OH kinase. Nature 2000, 407 (6803), 538–41. 10.1038/35035131. 11029009 PMC2670482

[ref8] Cavalcanti F. N.; Lucas T. F.; Lazari M. F.; Porto C. S. Estrogen receptor ESR1 mediates activation of ERK1/2, CREB, and ELK1 in the corpus of the epididymis. J. Mol. Endocrinol 2015, 54 (3), 339–49. 10.1530/JME-15-0086. 26069273

[ref9] Patel R.; Klein P.; Tiersten A.; Sparano J. A. An emerging generation of endocrine therapies in breast cancer: a clinical perspective. NPJ. Breast Cancer 2023, 9 (1), 20 10.1038/s41523-023-00523-4. 37019913 PMC10076370

[ref10] Rondon-Lagos M.; Villegas V. E.; Rangel N.; Sanchez M. C.; Zaphiropoulos P. G. Tamoxifen Resistance: Emerging Molecular Targets. Int. J. Mol. Sci. 2016, 17 (8), 1357 10.3390/ijms17081357. 27548161 PMC5000752

[ref11] Haddow A.; Watkinson J. M.; Paterson E.; Koller P. C. Influence of Synthetic Oestrogens on Advanced Malignant Disease. British Medical Journal 1944, 2 (4368), 393–398. 10.1136/bmj.2.4368.393. 20785660 PMC2286289

[ref12] Cole M. P.; Jones C. T. A.; Todd I. D. H. A New Anti-oestrogenic Agent in Late Breast Cancer: An Early Clinical Appraisal of ICI46474. Br. J. Cancer 1971, 25 (2), 270–275. 10.1038/bjc.1971.33. 5115829 PMC2008453

[ref13] Anderson G. L.; Limacher M.; Assaf A. R.; Bassford T.; Beresford S. A. A.; Black H.; Bonds D.; Brunner R.; Brzyski R.; Caan B.; Chlebowski R.; Curb D.; Gass M.; Hays J.; Heiss G.; Hendrix S.; Howard B. V.; Hsia J.; Hubbell A.; Jackson R.; Johnson K. C.; Judd H.; Kotchen J. M.; Kuller L.; LaCroix A. Z.; Lane D.; Langer R. D.; Lasser N.; Lewis C. E.; Manson J.; Margolis K.; Ockene J.; O’Sullivan M. J.; Phillips L.; Prentice R. L.; Ritenbaugh C.; Robbins J.; Rossouw J. E.; Sarto G.; Stefanick M. L.; Van Horn L.; Wactawski-Wende J.; Wallace R.; Wassertheil-Smoller S.; Women’s Health Initiative Steering C. Effects of conjugated equine estrogen in postmenopausal women with hysterectomy: the Women’s Health Initiative randomized controlled trial. JAMA 2004, 291 (14), 1701–1712. 10.1001/jama.291.14.1701. 15082697

[ref14] Menarche, menopause, and breast cancer risk: individual participant meta-analysis, including 118 964 women with breast cancer from 117 epidemiological studies. Lancet Oncol 2012, 13 (11), 1141–1151. 10.1016/S1470-2045(12)70425-4. 23084519 PMC3488186

[ref15] Pan K.; Lavasani S.; Aragaki A. K.; Chlebowski R. T. Estrogen therapy and breast cancer in randomized clinical trials: a narrative review. Menopause 2022, 29 (9), 1086–1092. 10.1097/GME.0000000000002021. 35969882

[ref16] Coser K. R.; Chesnes J.; Hur J.; Ray S.; Isselbacher K. J.; Shioda T. Global analysis of ligand sensitivity of estrogen inducible and suppressible genes in MCF7/BUS breast cancer cells by DNA microarray. Proc. Natl. Acad. Sci. U. S. A. 2003, 100 (24), 13994–9. 10.1073/pnas.2235866100. 14610279 PMC283534

[ref17] Lin C.-Y.; Strom A.; Vega V.; Li Kong S.; Li Yeo A.; Thomsen J. S; Chan W.; Doray B.; Bangarusamy D. K; Ramasamy A.; Vergara L. A; Tang S.; Chong A.; Bajic V. B; Miller L. D; Gustafsson J.-A.; Liu E. T Discovery of estrogen receptor alpha target genes and response elements in breast tumor cells. Genome Biol. 2004, 5 (9), R66 10.1186/gb-2004-5-9-r66. 15345050 PMC522873

[ref18] Yamaga R.; Ikeda K.; Horie-Inoue K.; Ouchi Y.; Suzuki Y.; Inoue S. RNA sequencing of MCF-7 breast cancer cells identifies novel estrogen-responsive genes with functional estrogen receptor-binding sites in the vicinity of their transcription start sites. Horm Cancer 2013, 4 (4), 222–32. 10.1007/s12672-013-0140-3. 23526455 PMC10358060

[ref19] Hah N.; Danko C. G.; Core L.; Waterfall J. J.; Siepel A.; Lis J. T.; Kraus W. L. A rapid, extensive, and transient transcriptional response to estrogen signaling in breast cancer cells. Cell 2011, 145 (4), 622–34. 10.1016/j.cell.2011.03.042. 21549415 PMC3099127

[ref20] Bourdeau V.; Deschenes J.; Laperriere D.; Aid M.; White J. H.; Mader S. Mechanisms of primary and secondary estrogen target gene regulation in breast cancer cells. Nucleic Acids Res. 2007, 36 (1), 76–93. 10.1093/nar/gkm945. 17986456 PMC2248750

[ref21] Lin C. Y.; Vega V. B.; Thomsen J. S.; Zhang T.; Kong S. L.; Xie M.; Chiu K. P.; Lipovich L.; Barnett D. H.; Stossi F.; Yeo A.; George J.; Kuznetsov V. A.; Lee Y. K.; Charn T. H.; Palanisamy N.; Miller L. D.; Cheung E.; Katzenellenbogen B. S.; Ruan Y.; Bourque G.; Wei C. L.; Liu E. T. Whole-genome cartography of estrogen receptor alpha binding sites. PLoS Genet 2007, 3 (6), e87 10.1371/journal.pgen.0030087. 17542648 PMC1885282

[ref22] Welboren W. J.; van Driel M. A.; Janssen-Megens E. M.; van Heeringen S. J.; Sweep F. C.; Span P. N.; Stunnenberg H. G. ChIP-Seq of ERalpha and RNA polymerase II defines genes differentially responding to ligands. EMBO J. 2009, 28 (10), 1418–28. 10.1038/emboj.2009.88. 19339991 PMC2688537

[ref23] Joseph R.; Orlov Y. L; Huss M.; Sun W.; Li Kong S.; Ukil L.; Fu Pan Y.; Li G.; Lim M.; Thomsen J. S; Ruan Y.; Clarke N. D; Prabhakar S.; Cheung E.; Liu E. T Integrative model of genomic factors for determining binding site selection by estrogen receptor-alpha. Mol. Syst. Biol. 2010, 6, 456 10.1038/msb.2010.109. 21179027 PMC3018168

[ref24] Carroll J. S.; Meyer C. A.; Song J.; Li W.; Geistlinger T. R.; Eeckhoute J.; Brodsky A. S.; Keeton E. K.; Fertuck K. C.; Hall G. F.; Wang Q.; Bekiranov S.; Sementchenko V.; Fox E. A.; Silver P. A.; Gingeras T. R.; Liu X. S.; Brown M. Genome-wide analysis of estrogen receptor binding sites. Nat. Genet. 2006, 38 (11), 1289–97. 10.1038/ng1901. 17013392

[ref25] Drabovich A. P.; Pavlou M. P.; Schiza C.; Diamandis E. P. Dynamics of Protein Expression Reveals Primary Targets and Secondary Messengers of Estrogen Receptor Alpha Signaling in MCF-7 Breast Cancer Cells. Mol. Cell Proteomics 2016, 15 (6), 2093–107. 10.1074/mcp.M115.057257. 27067054 PMC5083091

[ref26] Ong S. E.; Blagoev B.; Kratchmarova I.; Kristensen D. B.; Steen H.; Pandey A.; Mann M. Stable isotope labeling by amino acids in cell culture, SILAC, as a simple and accurate approach to expression proteomics. Mol. Cell Proteomics 2002, 1 (5), 376–86. 10.1074/mcp.M200025-MCP200. 12118079

[ref27] Zhu H.; Pan S.; Gu S.; Bradbury E. M.; Chen X. Amino acid residue specific stable isotope labeling for quantitative proteomics. Rapid Commun. Mass Spectrom. 2002, 16 (22), 2115–23. 10.1002/rcm.831. 12415544

[ref28] Liu L.; Zhou J.; Wang Y.; Mason R. J.; Funk C. J.; Du Y. Proteome alterations in primary human alveolar macrophages in response to influenza A virus infection. J. Proteome Res. 2012, 11 (8), 4091–101. 10.1021/pr3001332. 22709384 PMC3412919

[ref29] Tyanova S.; Temu T.; Cox J. The MaxQuant computational platform for mass spectrometry-based shotgun proteomics. Nat. Protoc 2016, 11 (12), 2301–2319. 10.1038/nprot.2016.136. 27809316

[ref30] Nguyen A. M.; Zhou J.; Sicairos B.; Sonney S.; Du Y. Upregulation of CD73 Confers Acquired Radioresistance and is Required for Maintaining Irradiation-selected Pancreatic Cancer Cells in a Mesenchymal State. Mol. Cell Proteomics 2020, 19 (2), 375–389. 10.1074/mcp.RA119.001779. 31879272 PMC7000112

[ref31] Tyanova S.; Cox J. Perseus: A Bioinformatics Platform for Integrative Analysis of Proteomics Data in Cancer Research. Methods Mol. Biol. 2018, 1711, 133–148. 10.1007/978-1-4939-7493-1_7. 29344888

[ref32] Cox J.; Mann M. MaxQuant enables high peptide identification rates, individualized p.p.b.-range mass accuracies and proteome-wide protein quantification. Nat. Biotechnol. 2008, 26 (12), 1367–72. 10.1038/nbt.1511. 19029910

[ref33] Subramanian A.; Tamayo P.; Mootha V. K.; Mukherjee S.; Ebert B. L.; Gillette M. A.; Paulovich A.; Pomeroy S. L.; Golub T. R.; Lander E. S.; Mesirov J. P. Gene set enrichment analysis: A knowledge-based approach for interpreting genome-wide expression profiles. Proc. Natl. Acad. Sci. U. S. A. 2005, 102 (43), 15545–15550. 10.1073/pnas.0506580102. 16199517 PMC1239896

[ref34] Huang D. W.; Sherman B. T.; Tan Q.; Collins J. R.; Alvord W. G.; Roayaei J.; Stephens R.; Baseler M. W.; Lane H. C.; Lempicki R. A. The DAVID Gene Functional Classification Tool: a novel biological module-centric algorithm to functionally analyze large gene lists. Genome Biol. 2007, 8 (9), R183 10.1186/gb-2007-8-9-r183. 17784955 PMC2375021

[ref35] Fonseka P.; Pathan M.; Chitti S. V.; Kang T.; Mathivanan S. FunRich enables enrichment analysis of OMICs datasets. J. Mol. Biol. 2021, 433 (11), 166747 10.1016/j.jmb.2020.166747. 33310018

[ref36] Zhou Z.; Zhou J.; Du Y. Estrogen receptor alpha interacts with mitochondrial protein HADHB and affects beta-oxidation activity. Mol. Cell Proteomics 2012, 11 (7), M111.011056-1 10.1074/mcp.M111.011056. PMC3394935 22375075

[ref37] Wlodkowic D.; Skommer J.; Darzynkiewicz Z. Flow cytometry-based apoptosis detection. Methods Mol. Biol. 2009, 559, 19–32. 10.1007/978-1-60327-017-5_2. 19609746 PMC3863590

[ref38] Badura M.; Braunstein S.; Zavadil J.; Schneider R. J. DNA damage and eIF4G1 in breast cancer cells reprogram translation for survival and DNA repair mRNAs. Proc. Natl. Acad. Sci. U. S. A. 2012, 109 (46), 18767–18772. 10.1073/pnas.1203853109. 23112151 PMC3503184

[ref39] Jian W.; Zhang X.; Wang J.; Liu Y.; Hu C.; Wang X.; Liu R. Scinderin-knockdown inhibits proliferation and promotes apoptosis in human breast carcinoma cells. Oncol Lett. 2018, 16 (3), 3207–3214. 10.3892/ol.2018.9009. 30127916 PMC6096137

[ref40] Yao G.; Lee T. J.; Mori S.; Nevins J. R.; You L. A bistable Rb–E2F switch underlies the restriction point. Nat. Cell Biol. 2008, 10 (4), 476–482. 10.1038/ncb1711. 18364697

[ref41] Rhodes D. R.; Yu J.; Shanker K.; Deshpande N.; Varambally R.; Ghosh D.; Barrette T.; Pandey A.; Chinnaiyan A. M. Large-scale meta-analysis of cancer microarray data identifies common transcriptional profiles of neoplastic transformation and progression. Proc. Natl. Acad. Sci. U. S. A. 2004, 101 (25), 9309–14. 10.1073/pnas.0401994101. 15184677 PMC438973

[ref42] Whitfield M. L.; George L. K.; Grant G. D.; Perou C. M. Common markers of proliferation. Nat. Rev. Cancer 2006, 6 (2), 99–106. 10.1038/nrc1802. 16491069

[ref43] Ohtani K.; Iwanaga R.; Nakamura M.; Ikeda M.; Yabuta N.; Tsuruga H.; Nojima H. Cell growth-regulated expression of mammalian MCM5 and MCM6 genes mediated by the transcription factor E2F. Oncogene 1999, 18 (14), 2299–309. 10.1038/sj.onc.1202544. 10327050

[ref44] Pedram A.; Razandi M.; Evinger A. J.; Lee E.; Levin E. R. Estrogen Inhibits ATR Signaling to Cell Cycle Checkpoints and DNA Repair. Mol. Biol. Cell 2009, 20 (14), 3374–3389. 10.1091/mbc.e09-01-0085. 19477925 PMC2710824

[ref45] Brunelli E.; Minassi A.; Appendino G.; Moro L. 8-Prenylnaringenin, inhibits estrogen receptor-alpha mediated cell growth and induces apoptosis in MCF-7 breast cancer cells. J. Steroid Biochem Mol. Biol. 2007, 107 (3–5), 140–8. 10.1016/j.jsbmb.2007.04.003. 17681752

[ref46] Paplomata E.; O’Regan R. The PI3K/AKT/mTOR pathway in breast cancer: targets, trials and biomarkers. Ther Adv. Med. Oncol 2014, 6 (4), 154–66. 10.1177/1758834014530023. 25057302 PMC4107712

[ref47] Xie D.; Pei Q.; Li J.; Wan X.; Ye T. Emerging Role of E2F Family in Cancer Stem Cells. Frontiers in Oncology 2021, 11, 723137 10.3389/fonc.2021.723137. 34476219 PMC8406691

[ref48] Schulze A.; Zerfass K.; Spitkovsky D.; Middendorp S.; Bergès J.; Helin K.; Jansen-Dürr P.; Henglein B. Cell cycle regulation of the cyclin A gene promoter is mediated by a variant E2F site. Proc. Natl. Acad. Sci. U.S.A. 1995, 92 (24), 11264–11268. 10.1073/pnas.92.24.11264. 7479977 PMC40612

[ref49] Prigent C.; Uzbekov R. Duplication and Segregation of Centrosomes during Cell Division. Cells 2022, 11 (15), 2445 10.3390/cells11152445. 35954289 PMC9367774

[ref50] Gibieza P.; Petrikaite V. The regulation of actin dynamics during cell division and malignancy. Am. J. Cancer Res. 2021, 11 (9), 4050–4069. 34659876 PMC8493394

[ref51] Yoshimura S. H.; Hirano T. HEAT repeats - versatile arrays of amphiphilic helices working in crowded environments?. J. Cell Sci. 2016, 129 (21), 3963–3970. 10.1242/jcs.185710. 27802131

[ref52] Koszegi Z.; Cheong R. Y. Targeting the non-classical estrogen pathway in neurodegenerative diseases and brain injury disorders. Frontiers in Endocrinology 2022, 13, 999236 10.3389/fendo.2022.999236. 36187099 PMC9521328

[ref53] Valverde R.; Edwards L.; Regan L. Structure and function of KH domains. FEBS J. 2008, 275 (11), 2712–26. 10.1111/j.1742-4658.2008.06411.x. 18422648

[ref54] Maris C.; Dominguez C.; Allain F. H. The RNA recognition motif, a plastic RNA-binding platform to regulate post-transcriptional gene expression. FEBS J. 2005, 272 (9), 2118–31. 10.1111/j.1742-4658.2005.04653.x. 15853797

[ref55] Hsu Y. T.; Wolter K. G.; Youle R. J. Cytosol-to-membrane redistribution of Bax and Bcl-X(L) during apoptosis. Proc. Natl. Acad. Sci. U. S. A. 1997, 94 (8), 3668–72. 10.1073/pnas.94.8.3668. 9108035 PMC20498

[ref56] Nechushtan A.; Smith C. L.; Hsu Y. T.; Youle R. J. Conformation of the Bax C-terminus regulates subcellular location and cell death. EMBO J. 1999, 18 (9), 2330–41. 10.1093/emboj/18.9.2330. 10228148 PMC1171316

[ref57] Wolter K. G.; Hsu Y. T.; Smith C. L.; Nechushtan A.; Xi X. G.; Youle R. J. Movement of Bax from the cytosol to mitochondria during apoptosis. J. Cell Biol. 1997, 139 (5), 1281–92. 10.1083/jcb.139.5.1281. 9382873 PMC2140220

[ref58] Song R. X. D.; Mor G.; Naftolin F.; McPherson R. A.; Song J.; Zhang Z.; Yue W.; Wang J.; Santen R. J. Effect of Long-Term Estrogen Deprivation on Apoptotic Responses of Breast Cancer Cells to 17 -Estradiol. JNCI Journal of the National Cancer Institute 2001, 93 (22), 1714–1723. 10.1093/jnci/93.22.1714. 11717332

[ref59] Hosford S. R.; Shee K.; Wells J. D.; Traphagen N. A.; Fields J. L.; Hampsch R. A.; Kettenbach A. N.; Demidenko E.; Miller T. W. Estrogen therapy induces an unfolded protein response to drive cell death in ER+ breast cancer. Mol. Oncol 2019, 13 (8), 1778–1794. 10.1002/1878-0261.12528. 31180176 PMC6670014

[ref60] Chen Q. Y.; Costa M. PI3K/Akt/mTOR Signaling Pathway and the Biphasic Effect of Arsenic in Carcinogenesis. Mol. Pharmacol. 2018, 94 (1), 784–792. 10.1124/mol.118.112268. 29769245 PMC5994485

[ref61] Brumatti G.; Salmanidis M.; Ekert P. G. Crossing paths: interactions between the cell death machinery and growth factor survival signals. Cell. Mol. Life Sci. 2010, 67 (10), 1619–1630. 10.1007/s00018-010-0288-8. 20157838 PMC11115775

[ref62] Abdallah M. E.; El-Readi M. Z.; Althubiti M. A.; Almaimani R. A.; Ismail A. M.; Idris S.; Refaat B.; Almalki W. H.; Babakr A. T.; Mukhtar M. H.; Abdalla A. N.; Idris O. F. Tamoxifen and the PI3K Inhibitor: LY294002 Synergistically Induce Apoptosis and Cell Cycle Arrest in Breast Cancer MCF-7 Cells. Molecules 2020, 25 (15), 3355 10.3390/molecules25153355. 32722075 PMC7436112

[ref63] Jiang H.; Fan D.; Zhou G.; Li X.; Deng H. Phosphatidylinositol 3-kinase inhibitor(LY294002) induces apoptosis of human nasopharyngeal carcinoma in vitro and in vivo. J. Exp. Clin. Cancer Res. 2010, 29 (1), 34 10.1186/1756-9966-29-34. 20412566 PMC2873422

[ref64] Altucci L.; Addeo R.; Cicatiello L.; Dauvois S.; Parker M. G.; Truss M.; Beato M.; Sica V.; Bresciani F.; Weisz A. 17beta-Estradiol induces cyclin D1 gene transcription, p36D1-p34cdk4 complex activation and p105Rb phosphorylation during mitogenic stimulation of G(1)-arrested human breast cancer cells. Oncogene 1996, 12 (11), 2315–2324. 8649771

[ref65] Foster J. S.; Wimalasena J. Estrogen regulates activity of cyclin-dependent kinases and retinoblastoma protein phosphorylation in breast cancer cells. Mol. Endocrinol. 1996, 10 (5), 488–498. 10.1210/mend.10.5.8732680. 8732680

[ref66] Bhukhai K.; Suksen K.; Bhummaphan N.; Janjorn K.; Thongon N.; Tantikanlayaporn D.; Piyachaturawat P.; Suksamrarn A.; Chairoungdua A. A phytoestrogen diarylheptanoid mediates estrogen receptor/Akt/glycogen synthase kinase 3beta protein-dependent activation of the Wnt/beta-catenin signaling pathway. J. Biol. Chem. 2012, 287 (43), 36168–78. 10.1074/jbc.M112.344747. 22936801 PMC3476284

[ref67] Asghar U.; Witkiewicz A. K.; Turner N. C.; Knudsen E. S. The history and future of targeting cyclin-dependent kinases in cancer therapy. Nat. Rev. Drug Discov 2015, 14 (2), 130–46. 10.1038/nrd4504. 25633797 PMC4480421

[ref68] Cicenas J.; Kalyan K.; Sorokinas A.; Jatulyte A.; Valiunas D.; Kaupinis A.; Valius M. Highlights of the Latest Advances in Research on CDK Inhibitors. Cancers (Basel) 2014, 6 (4), 2224–42. 10.3390/cancers6042224. 25349887 PMC4276963

[ref69] Yuan K.; Wang X.; Dong H.; Min W.; Hao H.; Yang P. Selective inhibition of CDK4/6: A safe and effective strategy for developing anticancer drugs. Acta Pharm. Sin B 2021, 11 (1), 30–54. 10.1016/j.apsb.2020.05.001. 33532179 PMC7838032

[ref70] Dhanasekaran R.; Deutzmann A.; Mahauad-Fernandez W. D.; Hansen A. S.; Gouw A. M.; Felsher D. W. The MYC oncogene — the grand orchestrator of cancer growth and immune evasion. Nature Reviews Clinical Oncology 2022, 19 (1), 23–36. 10.1038/s41571-021-00549-2. PMC9083341 34508258

[ref71] Wang C.; Mayer J. A.; Mazumdar A.; Fertuck K.; Kim H.; Brown M.; Brown P. H. Estrogen Induces c-myc Gene Expression via an Upstream Enhancer Activated by the Estrogen Receptor and the AP-1 Transcription Factor. Mol. Endocrinol. 2011, 25 (9), 1527–1538. 10.1210/me.2011-1037. 21835891 PMC3165912

[ref72] Hoffman B.; Liebermann D. A. Apoptotic signaling by c-MYC. Oncogene 2008, 27 (50), 6462–6472. 10.1038/onc.2008.312. 18955973

[ref73] Mitchell K. O.; Ricci M. S.; Miyashita T.; Dicker D. T.; Jin Z.; Reed J. C.; El-Deiry W. S. Bax is a transcriptional target and mediator of c-myc-induced apoptosis. Cancer Res. 2000, 60 (22), 6318–25. 11103792

[ref74] Juin P.; Hueber A. O.; Littlewood T.; Evan G. c-Myc-induced sensitization to apoptosis is mediated through cytochrome c release. Genes Dev. 1999, 13 (11), 1367–81. 10.1101/gad.13.11.1367. 10364155 PMC316765

