# Supplementary material for: Proteomic Analysis Reveals Major Proteins and Pathways That Mediate the Effect of 17-β-Estradiol in Cell Division and Apoptosis in Breast Cancer MCF7 Cells
Source: J Proteome Res. 2024 Oct 11;23(11):4835–48. doi: 10.1021/acs.jproteome.4c00102 (PMC11536429; doi:10.1021/acs.jproteome.4c00102)
Supplement: Supplementary file 1 — pr4c00102_si_002.pdf [file pr4c00102_si_002.pdf]

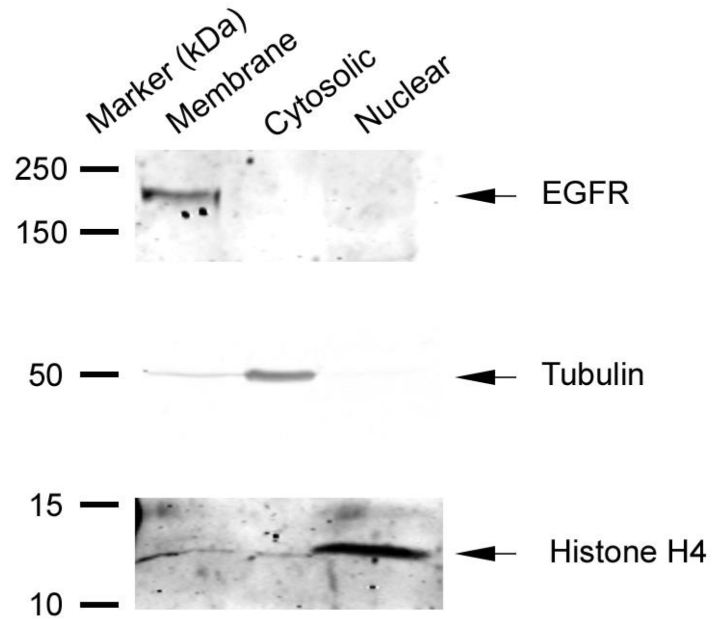

Supporting Figure S1. Fractionation of cellular proteins into cytosolic, nuclear, and membrane/organelle fractions. MCF7 cells were fractionated into cytosolic, nuclear, and membrane/organelle fractions using differential centrifugation. The fractionated proteins were then analyzed by Western blotting using the indicated antibodies. An antibody specifically recognizing the plasma membrane protein epidermal growth factor receptor (EGFR) was used as a marker for membrane proteins, while anti-tubulin and anti-histone H4 antibodies were used as markers for cytosolic and nuclear proteins, respectively.

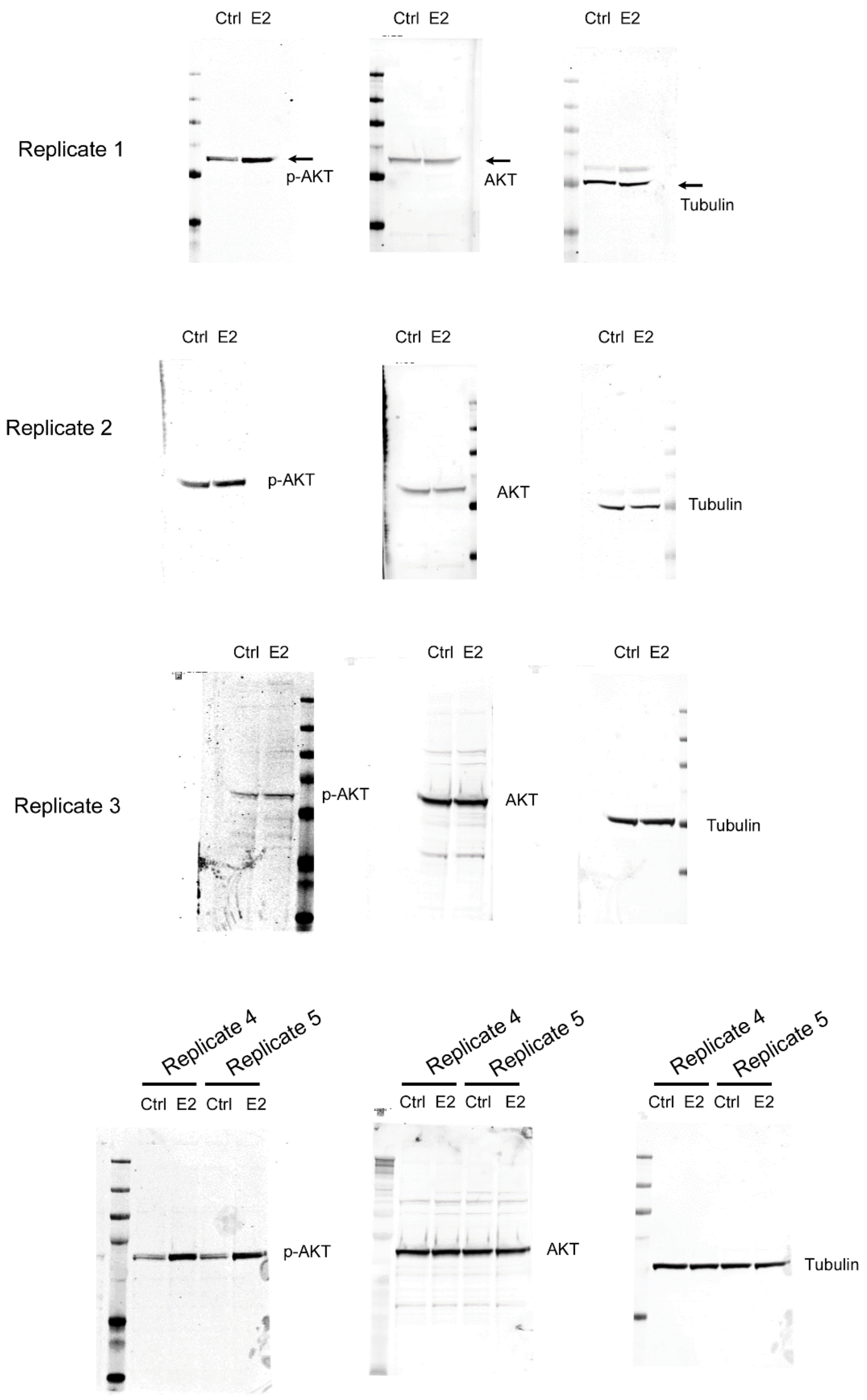

Supporting Figure S2. Uncropped images of the Western blots showing AKT Ser-473 phosphorylation in E2-treated MCF7 cells (E2) compared to vehicle-treated control cells (Ctrl). Five independent replicates (n=5) of Western blot analyses were performed using separately prepared biological samples. The biological samples for replicates 4 and 5 were prepared independently but were run on the same gel.

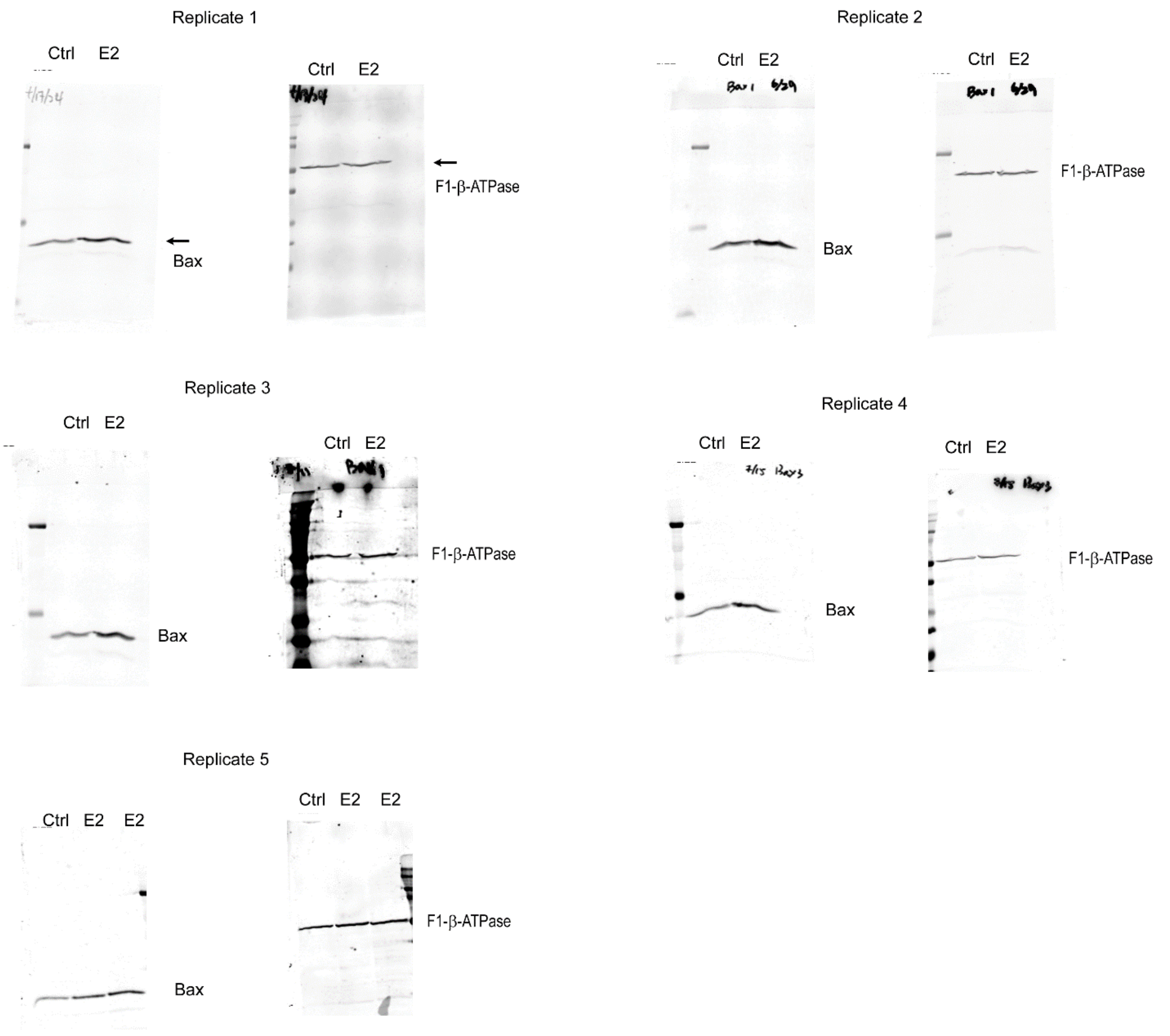

Supporting Figure S3. Uncropped images of the Western blots showing elevated Bax protein levels in the membrane/organelle fraction of E2-treated MCF7 cells (E2) compared to vehicle-treated control cells (Ctrl). The five independent replicates (n=5) of Western blot analyses were performed using separately prepared biological samples.
