# Supplementary material for: Proteomic Analysis Reveals Major Proteins and Pathways That Mediate the Effect of 17-β-Estradiol in Cell Division and Apoptosis in Breast Cancer MCF7 Cells
Source: J Proteome Res. 2024 Oct 11;23(11):4835–48. doi: 10.1021/acs.jproteome.4c00102 (PMC11536429; doi:10.1021/acs.jproteome.4c00102)
Supplement: Supplementary file 9 — pr4c00102_si_010.pdf [file pr4c00102_si_010.pdf]

Supporting Table S8. Functional annotation analysis by DAVID of the proteins whose expression was significantly downregulated by E2: with GOTERM_BP_DIRECT and GOTERM_MF_DIRECT.*

**Cluster 1**	**Enrichment Score: 2.36**				
**Category**	**Term**	**Count**	**UniProt ID**	***p* Value**	**Fold Enrichment**
GOTERM_MF _DIRECT	GO:0051015: actin filament binding	8	P35580, Q15149, Q14847, Q9Y6U3, O43795, Q9BPX5, P08133, P35579	4.76E: 05	8.3
GOTERM_BP _DIRECT	GO:0030048: actin filament-based movement	3	P35580, O43795, P35579	0.0021	42.8
GOTERM_MF _DIRECT	GO:0003774: motor activity	4	P42765, P35580, O43795, P35579	0.0022	15.3
GOTERM_MF _DIRECT	GO:0000146: microfilament motor activity	3	P35580, O43795, P35579	0.0104	19.2
GOTERM_MF _DIRECT	GO:0003779: actin binding	6	P42765, P35580, Q15149, Q9Y6U3, O43795, P35579	0.0139	4.2

* Only the categories and terms with $p < 0.05$ are listed.
