# Supplementary material for: Proteomic Analysis Reveals Major Proteins and Pathways That Mediate the Effect of 17-β-Estradiol in Cell Division and Apoptosis in Breast Cancer MCF7 Cells
Source: J Proteome Res. 2024 Oct 11;23(11):4835–48. doi: 10.1021/acs.jproteome.4c00102 (PMC11536429; doi:10.1021/acs.jproteome.4c00102)
Supplement: Supplementary file 10 — pr4c00102_si_011.pdf [file pr4c00102_si_011.pdf]

Supporting Table S9. Functional annotation analysis by DAVID of the proteins whose expression was significantly downregulated by E2: with UP_SEQ_FEATURE.*

Category	Term	Count	UniProt ID	*p* Value	Fold Enrichment
**Cluster 1**	**Enrichment Score: 1.44**				
UP_SEQ_FEATURE	DOMAIN:RRM	5	Q16629, P52272, Q9UHX1, P84103, P26368	0.013	5.4

* Only the categories and terms with $p < 0.05$ are listed.
